# Intranasally delivered mesenchymal stromal cells decrease glial inflammation early in prion disease

**DOI:** 10.3389/fnins.2023.1158408

**Published:** 2023-05-12

**Authors:** Arielle J. D. Hay, Amanda S. Latham, Genova Mumford, Amelia D. Hines, Sydney Risen, Elizabeth Gordon, Connor Siebenaler, Vincenzo S. Gilberto, Mark D. Zabel, Julie A. Moreno

**Affiliations:** ^1^Prion Research Center, Department of Microbiology, Immunology, and Pathology, College of Veterinary Medicine and Biomedical Sciences, Colorado State University, Fort Collins, CO, United States; ^2^Department of Environmental and Radiological Health Sciences, College of Veterinary Medicine and Biomedical Sciences, Colorado State University, Fort Collins, CO, United States; ^3^Brain Research Center, College of Veterinary Medicine and Biomedical Sciences, Colorado State University, Fort Collins, CO, United States; ^4^Center for Healthy Aging, College of Veterinary Medicine and Biomedical Sciences, Colorado State University, Fort Collins, CO, United States

**Keywords:** microglia, astrocytes, prion, inflammation, cytokines, mesenchymal stromal cells

## Abstract

Mesenchymal stromal cells (MSCs) are an intriguing avenue for the treatment of neurological disorders due to their ability to migrate to sites of neuroinflammation and respond to paracrine signaling in those sites by secreting cytokines, growth factors, and other neuromodulators. We potentiated this ability by stimulating MSCs with inflammatory molecules, improving their migratory and secretory properties. We investigated the use of intranasally delivered adipose-derived MSCs (AdMSCs) in combating prion disease in a mouse model. Prion disease is a rare, lethal neurodegenerative disease that results from the misfolding and aggregation of the prion protein. Early signs of this disease include neuroinflammation, activation of microglia, and development of reactive astrocytes. Later stages of disease include development of vacuoles, neuronal loss, abundant aggregated prions, and astrogliosis. We demonstrate the ability of AdMSCs to upregulate anti-inflammatory genes and growth factors when stimulated with tumor necrosis factor alpha (TNFα) or prion-infected brain homogenates. We stimulated AdMSCs with TNFα and performed biweekly intranasal deliveries of AdMSCs on mice that had been intracranially inoculated with mouse-adapted prions. At early stages in disease, animals treated with AdMSCs showed decreased vacuolization throughout the brain. Expression of genes associated with Nuclear Factor-kappa B (NF-κB) and Nod-Like Receptor family pyrin domain containing 3 (NLRP3) inflammasome signaling were decreased in the hippocampus. AdMSC treatment promoted a quiescent state in hippocampal microglia by inducing changes in both number and morphology. Animals that received AdMSCs showed a decrease in both overall and reactive astrocyte number, and morphological changes indicative of homeostatic astrocytes. Although this treatment did not prolong survival or rescue neurons, it demonstrates the benefits of MSCs in combatting neuroinflammation and astrogliosis.

## 1. Introduction

Prion diseases are fatal neurodegenerative diseases characterized by the misfolding and aggregation of the prion protein (PrP), due to either a genetic mutation in the protein, or a spontaneous or acquired source of misfolded PrP (denoted PrP^Sc^) (Prusiner, 1982). According to the CDC, each year approximately 1.5 per one million people in the United States develop classic Creutzfeldt-Jakob Disease (CJD), the most common spontaneous prion disease (CDC, 2021). Individuals with CJD undergo rapid loss of memory and motor function, which ultimately lead to death. There are currently no available treatments for CJD or any other prion diseases, because after clinical signs occur, PrP^Sc^ accumulation and neurodegeneration are irreversible.

An early sign of prion disease, prior to detectable PrP^Sc^ in the brain or behavioral changes, is astrogliosis. In animal models, astrocyte numbers increase throughout the brain prior to signs of neurodegeneration. This is accompanied by an increase in microglia numbers, as well as inflammatory cytokines and chemokines (Sandberg et al., 2014; Carroll and Chesebro, 2019; Carroll et al., 2020). There is evidence that PrP^Sc^ aggregates are not the only source of neurotoxicity, and that neuroinflammation may be contributing to neuronal loss and degeneration (Giese et al., 1998; Garcao et al., 2006; Liddelow et al., 2017; Smith et al., 2020; Lakkaraju et al., 2022). An ideal treatment should therefore target both early inflammation as well as protein aggregation.

Here, we investigate the therapeutic potential of adipose-derived mesenchymal stromal cells (AdMSCs) in decreasing glial inflammation in a mouse model of prion disease. These cells can be easily isolated from adipose tissue, expanded in culture, and injected into animals or patients with little immunogenic effects (Li et al., 2022; Sanchez-Castillo et al., 2022). They follow chemokine gradients to migrate to sites of inflammation, where they respond by secreting anti-inflammatory cytokines, chemokines, and growth factors (Song et al., 2011; Xiao et al., 2012; Fu et al., 2019; Li et al., 2022). We recently demonstrated the therapeutic ability of AdMSCs in decreasing glial inflammation in a cell-culture model of prion disease through a reduction of genes associated with signaling pathways such as pro-inflammatory transcription factor, Nuclear Factor-kappa B (NF-κB) and the Nod-Like Receptor family pyrin domain containing 3 (NLRP3) inflammasome (Hay et al., 2022). MSCs also decrease markers of reactive astrocytes and M1 activated microglia (Shan et al., 2017; Liu et al., 2019, 2021; Tang et al., 2021; Hay et al., 2022), cell types which are highly abundant in prion infection. Mesenchymal stromal cells (MSCs) have been used in mouse models of neurodegenerative diseases such as Alzheimer’s and Parkinson’s (Chen et al., 2017, 2020; Hu and Wang, 2021; Peng et al., 2022). MSCs derived from bone marrow and compact bone have been used to decrease inflammation in prion-diseased mice when delivered intracranially through stereotaxic surgery (Song et al., 2009; Shan et al., 2017). Although this was successful in increasing the lifespan of these animals, it poses some limitations when translated to the clinic, as acquiring MSCs from the bone and injecting them stereotaxically into the brain are very invasive procedures.

In this study, we investigate the effectiveness of intranasal delivery of MSCs from adipose tissue into mice with prion disease. We delivered cytokine-stimulated AdMSCs derived from prion knockout (PrP KO) mice every 2 weeks beginning at 10 weeks post-infection (wpi) and ending at 20 wpi. We assessed both behavioral and clinical signs of disease, neuronal loss and vacuolization, development of inflammation, and astrogliosis as disease progressed.

## 2. Materials and methods

### 2.1. Animal care and ethics statement

All mice were bred and maintained at Colorado State’s Lab Animal Resources, accredited by the Association for Assessment and Accreditation of Lab Animal Care International. This was done in accordance with protocols approved by the Institutional Animal Care and Use Committee at Colorado State University.

### 2.2. Brain preparations

C57Bl/6 (Jackson Laboratory) mice were anaesthetized with isoflurane prior to intracranial inoculation with 30 μl of 1% Rocky Mountain Laboratories (RML) strains of mouse-adapted prions, or normal brain homogenate (NBH). Mice were subjected to intranasal delivery of AdMSCs and behavioral assays, as described below. Mice were euthanized for time-course study at 14, 16, and 18 wpi, 3 days after intranasal delivery was performed, while the remaining mice were taken to terminal disease. Mice were monitored for weight loss and clinical signs of prion disease and euthanized by deep isoflurane anaesthetization followed by decapitation after showing signs of terminal illness. NBH mice were sacrificed after all prion-infected mice had been euthanized. The right hemisphere was fixed in 10% neutral-buffered formalin. The left hemisphere was removed and olfactory bulbs, hippocampus, thalamus and cerebellum were removed and stored in RNAlater (Qiagen) at –80°C prior to RNA analysis. The remainder of the left hemisphere was used to make 20% brain homogenates in phosphate-buffered saline (PBS) using beads and a tissue homogenizer (Benchmark Bead Blaster 24) and stored at –80°C prior to western blot analysis.

### 2.3. Isolating and maintaining AdMSCs

Adipose-derived MSCs were isolated as described previously (Hay et al., 2022). Adult TALEN PrP knock-out (KO) C57Bl/6 mice (Nuvolone et al., 2016) were euthanized and abdominal adipose tissue was removed and placed in Hank’s Buffered Saline Solution containing 25% Trypsin (HyClone, 0.25%). Adipose tissue was dissociated by incubating with a mixture of 200 U/ml DNase-I (Roche) and 400 U/ml Stemxyme (Worthington Biochemical Corporation) in DMEM/F12 media (Caisson Labs) at 37°C for 1.5 h. The tissues were triturated and centrifuged at 4°C for 5 min at 1000 × *g* to pellet the stromal vascular fraction. The pellet was washed once with sterile PBS and centrifuged at 1000 × *g*. The pellet was resuspended in 1 ml of AdMSC media (low glucose DMEM containing l-glutamine and supplemented with essential and non-essential amino acids (Gibco), 15% heat-inactivated fetal bovine serum (FBS) (Peak Serum), and penicillin/streptomycin/neomycin (PSN) (Sigma)). Resuspension was filtered through a 40 μm cell strainer (Fisher) to remove any non-dissociated tissue. Cells were plated in 10 cm dishes and grown in AdMSC media at 37° C with 5% CO_2_. Seventy-two hours later, cells were passaged at a 1:3 ratio and again every 3–4 days.

### 2.4. Stimulating AdMSCs for mRNA transcript analysis

Adipose-derived mesenchymal stromal cells were plated at 100,000 cells per well in 6 cm plates at passage 3. The following day, media was removed and replaced with media containing 10 ng/ml tumor necrosis factor alpha (TNFα), 100 ng/ml interferon gamma (IFNγ), or normal media as a negative control. Twenty-four hour later, cells were washed twice with sterile PBS and cell lysates were obtained using RLT buffer (Qiagen) containing β-mercaptoethanol (Sigma-Aldrich) and filtered through a QiaShredder column (Qiagen). RNA isolation and qRT-PCR protocol is described below.

### 2.5. Intranasal delivery of AdMSCs

PrP KO AdMSCs at passage 3 were stimulated with 10 ng/ml TNFα 24 h prior to intranasal delivery. Cells were washed three times with sterile PBS and trypsinized with 0.25% Trypsin, resuspended in AdMSC media, then spun at 4C for 5 min at 1000 × *g*. Cells were washed thoroughly with PBS, spun an additional time, counted on a hemocytometer, and resuspended in PBS at 1×10^6^ cells per 18 μl. Mice were anaesthetized with isoflurane and treated with 100 U hyaluronidase (United States Biochemical Corporation) in PBS, with 3 μl delivered to each nostril, three times per nostril, for a total of 18 μl, 1 h prior to intranasal AdMSC delivery. Three μl AdMSC cell suspension was then delivered to each nostril, three times per nostril, for a total of 18 μl, or 1×10^6^ cells per animal. Control mice were given PBS containing no AdMSCs. Mice were monitored for 10 min after regaining consciousness to ensure no adverse side effects. AdMSC treatments were performed at 10, 12, 14, 16, 18, and 20 weeks post infection (wpi). This experiment was repeated twice. The first cohort contained all female mice, 12 NBH mice and 24 of RML-infected (16 received AdMSCs and 12 received PBS), all of which were taken to terminal stages of disease. The second cohort contained both male and female mice, 7 NBH mice and 40 RML mice (24 received AdMSCs and 16 received PBS). 10 mice (6 AdMSC and 4 PBS) were euthanized at 14, 16, and 18 wpi.

### 2.6. Clinical and behavioral assays

Ability of mice to build nests was evaluated by placing three fresh napkins weekly in the cage. Twenty-four hour later, the position of the napkins was evaluated on a score of 0–5, with 0 being untouched, dirty and marked with urine, and 5 being positioned into a compact nest that provided full shelter to the mice. Nests were evaluated beginning at 10 wpi until euthanasia. For burrowing assessment, female mice were separated into individual cages containing a 6-inch section PVC pipe that was closed off at one end and filled with 120 grams of food pellets. Mice were given 30 min to “burrow” into the PVC pipe by removing the pellets. Mice were returned to their home cage and the remaining pellets in the PVC pipe were weighed. Burrowing was performed on mice every other week beginning at week 13 and ending at week 21. Clinical signs and weight were evaluated beginning at 17 wpi and continuing until mice were euthanized. Mice were evaluated weekly and scored from 0 (no signs) to 2 (severe signs) on the following signs of RML prion disease: tail rigidity, hyperactivity, ataxia, extensor reflex, tremors, righting reflex, kyphosis and poor grooming. Mice were additionally monitored for severe weight loss. Clinical signs and weight were monitored twice a week beginning at 20 wpi. When mice reached a total score of 10 for any combination of signs they were euthanized. Mice inoculated with normal brain homogenate (NBH) were used as a control for all behavioral assays and clinical signs.

### 2.7. Immunohistochemistry

Fixed brain hemispheres were embedded in paraffin and sliced and mounted on slides at 5 μm using the HM325 microtome (Thermo Scientific). Tissue was deparaffinized and rehydrated and underwent antigen retrieval in 0.01 M sodium citrate buffer for 20 min at 95°C. Endoperoxidases were inactivated by incubating tissue in 0.3% hydrogen peroxide. Tissue was blocked in Tris A [Tris-buffered saline (TBS) and Triton-X (Sigma-Aldrich)] containing 2% bovine serum albumin (BSA, Sigma-Aldrich) and 10% horse serum (Corning) for 1 h at room temperature. Primary antibodies were made in Tris A/2% BSA and incubated overnight in a humidity chamber at 4° C. The following primary antibodies were used: Iba1 (Abcam) at 1:400 dilution and GFAP (Dako) at 1:400 dilution. Tissue was washed with Tris A/2% BSA and incubated with biotinylated secondary antibody at 1:250 (Vector Laboratories) for 1 h, washed, and incubated with ABC complex (Vector Laboratories) for 1 h as per manufacturer’s instructions, washed, and incubated with DAB (Vector Laboratories) until color change was observed (time dependent on antibody used). Tissue was washed with TBS and counterstained with hematoxylin (Epredia) and bluing reagent (Cancer Diagnostics, Inc). Tissue was dehydrated and coverslips (Globe, #1) were mounted with media (Epredia) and dried overnight or longer before imaging with the Olympus VS120 Scanning Microscope. Representative 40x images were taken using the Olympus BX53. Cell counts were performed using the Olympus cellSens software (v 1.18). Outliers were identified and removed using a ROUT outlier test and a Welch’s T-test was used to compare treated and untreated groups using Prism (v 9.1.0).

### 2.8. Immunofluorescence quantification and skeletonization

Tissue was deparaffinized, rehydration, sodium citate treatment, and blocked in Tris A/BSA and incubated overnight, as described above. The following primary antibodies were used: Iba1 (Abcam) at 1:50 dilution, GFAP (Dako) at 1:100 dilution, S100β (Abcam) at 1:750 dilution and C3 (Abcam) at 1:250. Tissue was washed with TBS and incubated for 1 h in the dark with Alexa Fluor-488, −555 or −647 (Invitrogen) secondary antibodies at 1:500 dilution and 2% normal donkey serum (Jackson ImmunoResearch). Slides were washed and incubated in Hoechst at 1:2000 dilution in PBS for 3 min. Slides were coverslipped with Prolong Gold Antifade mounting media (Thermo Scientific) and kept in the dark at room temperature for 24 h, then in 4°C prior to imaging. GFAP slides were imaged on an Olympus BX60 fluorescent scope with a DP23 camera. For skeletonization experiments, four to five regions between the dentate gyrus and CA1-CA3 region of the hippocampus were imaged at 40x for each animal. All other slides were imaged with an Olympus BX63 fluorescence microscope equipped with a motorized stage and Hamamatsu ORCA-flash 4.0 LT CCD camera and an Olympus Xline apochromat 20X (0.8 N.A.) air objective. Exposures for each stain were set for the same period of time within each channel. Regions of interest (ROI) were selected with Olympus cellSens software to identify S100β^+^ astrocytes within the hippocampus using adaptive thresholding and then converted into individual ROIs. Mean gray intensity of C3 was determined within each S100β^+^ cell ROI. Skeletonization of astrocytes and microglia was performed using IMARIS 9.9.1. Using the calculate soma model, new starting points were detected using the largest diameter of 9.5 um, and seed points using the thinnest diameter of 0.570 um. Astrocytes were analyzed in channel 555 with a manual starting point threshold at 33.3, and seed point threshold at 43.1. Microglia were analyzed in channel 647 with automatic starting point threshold and seed point thresholds. These numbers were adjusted for each image to eliminate background. Seed points around starting points were removed using a sphere region diameter of 19.0 um. Disconnected segments were removed with an automatic absolute intensity threshold for both astrocytes and microglia. The dendrite diameter threshold was set at 4.51 for astrocytes and 4.91 for microglia. A max gap length of 11.4 um was used. After filaments were traced, processes that did not correspond to fluorescent staining were manually removed. Data generated by IMARIS was analyzed in Prism (v 9.1.0). Outliers were identified and removed using a ROUT outlier test and a Welch’s T-test was used to compare treated and untreated groups.

### 2.9. Hematoxylin and eosin staining

Slides were deparaffinized and rehydrated before being treated with hematoxylin and bluing reagent. Tissue was counterstained with eosin (Epredia). Slides were dehydrated and coverslips (Globe, #1) were mounted with media. Slides were imaged with the Olympus VS120 Scanning Microscope. Vacuoles were scored for each brain region (frontal cortex, hippocampus, thalamus and cerebellum) on a scale of 0 (no pathology) to 5 (significant pathology) by three pathologists who were blinded to the treatment groups (Fraser and Dickinson, 1968; Scott et al., 1997; Sun et al., 2023). Both quantity and size of vacuoles were considered during scoring. An average of the three scores was taken for each brain region. Pyknotic neurons were counted manually within the CA1 region of the hippocampus.

### 2.10. Immunoblotting

Twenty percent brain homogenates were analyzed with a BCA Protein Assay Kit (Thermo Scientific) to quantify protein concentration. Hundred μg of protein was digested with a final volume of 20 μg /ml proteinase K (PK) (Roche) for PrP^Sc^ blots for 1 h at 37°C. Digestion was terminated with 2 mM PMSF (Thermo Fisher) and samples were suspended in loading buffer (Bio-Rad) containing *β*-mercaptoethanol (Sigma-Aldrich) before being loaded on a gel. For PrP^C^ blots, 25 μg of sample was used with no PK digestion. Samples were run on a 4–20% acrylamide SDS page gels (Bio-Rad) and transferred to PVDF blotting paper (MilliPore). Primary antibody Bar-224 (Cayman Chemical Company) was used at 1:10,000 dilution. HRP-conjugated secondary antibodies were used at a concentration of 1:5,000 (Vector Laboratories). After imaging, PrP^C^ blots were stripped and loading control GAPDH was ran at a 1:10,000 dilution (MilliPore), with HPR-conjugated secondary antibody at 1:5,000 dilution (Southern Biotech). Western blots were visualized using SuperSignal West Pico PLUS Chemiluminescent Substrate (Thermo Scientific) and visualized with the BioRad ChemiDoc MP.

### 2.11. Live animal imaging

Forty-eight CD-1 mice (Charles River) were inoculated intracranially with 1% RML, as described above. PrP KO AdMSCs were isolated and expanded as described above, stimulated for 24 h with 10 ng/ml TNFα, then labeled with DiD Vybrant Cell-Labeling Solution (Invitrogen) (Garrovo et al., 2013; Donega et al., 2014), following manufacturer’s protocol, or mock-labeled with PBS. Cells were intranasally delivered to the CD-1 mice at 18 weeks post-infection, as described above. Twenty-four mice received unlabeled cells, the remaining 24 received labeled cells. Mice were imaged with a Xenogen IVIS Imaging System at 24, 48, 72 h, 7 days and 14 days post-delivery. At each timepoint, brains were extracted from 4 mice receiving both labeled AdMSCs and 4 mice receiving unlabeled AdMSCs. Brains were fixed for 60 s in 10% neutral buffered formalin (NBF) and then imaged with the Xenogen IVIS with an excitation filter of 640 and an emission filter of 680. Maximum radiance efficiency was measured for both animals and brains by creating a region of interest (ROI). The same size ROI was used to analyze each animal or brain for each image.

This study was repeated in a cohort of 12 RML-infected female CD-1 mice that did not undergo live-animal imaging, to prevent any loss of signal from the DiD-labeled AdMSCs. Ten mice received DiD-labeled AdMSCs, and the remaining 2 received mock-labeled AdMSCs. Six mice were euthanized at 48 h post-delivery (one control and 5 DiD-labeled), and the remaining 6 were euthanized at 7 days post-delivery, and brains were bisected sagitally, hemispheres were placed side-by-side in OCT Freezing Medium (Sakura Finetek) and immediately frozen on dry ice. Brains were cut sagitally using a Microm HM 525 Cryostat in 10 μm-thick sections. Each slide received 3 sets of sections (each consisting of both hemispheres), with 50 μm separation between each set. 10 slides were made for each brain, spanning approximately 1,500 μm from the most medial section to the most lateral section. The first (most medial), middle and last (most lateral) slides were selected, washed with PBS, incubated in Hoechst at 1:2000 dilution in PBS for 3 min, and coverslipped with Prolong Gold Antifade mounting media (Thermo Scientific) and kept in the dark at room temperature for 24 h, then in 4°C prior to imaging. Slides were imaged with an Olympus BX63 fluorescence microscope equipped with a motorized stage and Hamamatsu ORCA-flash 4.0 LT CCD camera and an Olympus Xline apochromat 20X (0.8 N.A.) air objective. DiD (Cy5) exposure time was selected based on control slides. For each brain, the cortex, hippocampus, thalamus and cerebellum were analyzed and presence of DiD+ cells were recorded. Representative images for each brain region showing significant cell migration were taken at 20x.

Slides from brains that showed significant DiD+ cells in the hippocampus were selected and blocked in Tris A/BSA and incubated overnight, as described above. The following primary antibodies were used: Oct3/4 (Abcam) at 1:100 dilution, Vimentin (Abcam) at 1:100 dilution. Tissue was washed with TBS and secondary antibody at 1:500 (Southern Biotech) containing 2% serum was incubated at 1:500 dilution in the dark for 1 h at room temperature. Slides were washed, counterstained with Hoechst, and coverslipped. Hippocampi were imaged at 20x to identify DiD+ cells that also expressed Oct3/4 and Vimentin, indicative of AdMSCs.

### 2.12. Reverse transcriptase quantitative PCR analysis

RNA was extracted from cells or hippocampal tissue using QIAshredder and RNeasy extraction kits, in accordance with manufacturer’s protocol, including a DNase digestion step with the RNase free DNase kit (Qiagen, Valencia, CA, United States). Purity and concentration were determined using a ND-1000 spectrophotometer (NanoDrop Technologies, Wilmington, DE). 25–50 ng RNA was reverse transcribed using the iScript Reverse Transcriptase kit (BioRad, Hercules CA). The cDNA was amplified within 24 h of reverse transcription using iQ SYBR Green Supermix (BioRad, Hercules CA). The corresponding validated primer sequences were used for each gene at 10 mM. The expression data was analyzed using the 2^–ΔΔCT^ method and normalized to expression of reference gene *β-actin* (Livak and Schmittgen, 2001). All RT-PCR was done following MIQE guidelines. Validated primer sequences are listed in Table 1.

**Table 1:** Primer sequences for reverse transcriptase quantitative PCR.

Gene	Forward primer	Reverse primer
TSG-6	GCTACAACCCACATGCAAAGGA	CCGTACTTGAGCCGAATGTGC
TGFβ1	CTTCAATACGTCAGACATTCGGG	GTAACGCCAGGAATTGTTGCT
VEGF	ACTTTCTGCTCTCTTGGGTGC	GCAGCCTGGGACCACTTG
FGF1	AAAGTGCGGGCGAAGTGTAT	CTCATTTGGTGTCTGCGAGC
IL1β	GCAGCAGCACATCAACAAG	CACGGGAAAGACACAGGTAG
CCL5	TTAAAAACCTGGATCGGAACCAA	TCGAGTGACAAACACGACTGC
TNFα	CCGATGGGTTGTACCTTGTC	AGATAGCAAATCGGCTGACG
C3	GAGCGAAGAGACCATCGTACT	TCTTTAGGAAGTCTTGCACAGTG
C1qa	AGAGAGGGGAGCCAGGAGC	CATTGCCAGGTTTGCCAGGG
NLRP3	CCTGGGGGACTTTGGAATCA	GACAACACGCGGATGTGAGA
Caspase-1	AACCACTCGTACACGTCTTGC	ATCCTCCAGCAGCAACTTCA
IL18	GACTCTTGCGTCAACTTCAAGG	GTTGTCTGATTCCAGGTCTCCA
CD16	TTTGGACACCCAGATGTTTCAG	GTCTTCCTTGAGCACCTGGATC
CD32	AATCCTGCCGTTCCTACTGATC	GTGTCACCGTGTCTTCCTTGAG
IL-6	CTGCAAGAGACTTCCATCCAG	AGTGGTATAGACAGGTCTGTTGG
CCL2	TTAAAAACCTGGATCGGAACCAA	GCATTAGCTTCAGATTTACGGGT
iNos	CCCTTCAATGGTTGGTACATGG	ACATTGATCTCCGTGACAGCC
NF-κB1	GTGGAGGCATGTTCGGTAGT	CCTGCGTTGGATTTCGTGAC
Arg-1	CGTAGACCCTGGGGAACACTAT	TCCATCACCTTGCCAATCCC
Igf-1	AAAGCAGCCCGCTCTATCC	CTTCTGAGTCTTGGGCATGTCA
*β*-actin	GCTGTGCTATGTTGCTCTAG	CGCTCGTTGCCAATAGTG

### 2.13. Statistical analysis

For all analyses, outliers were identified and removed using a ROUT outlier test (*Q* = 1%). Cleaned data for two groups was measured using a Welch’s *T*-test. For three or more groups, cleaned data was analyzed using a One-way ANOVA with Tukey’s *post hoc* analysis. A value of *p* of 0.05 was used for all analyses. All figures present mean +/− standard error of the mean (SEM). All data analysis and generation of graphs was done with Prism (v 9.1.0).

## 3. Results

### 3.1. Fluorescently-labeled AdMSCs migrate deep into the olfactory system and into the brains of prion-infected mice

**Figure 1:**
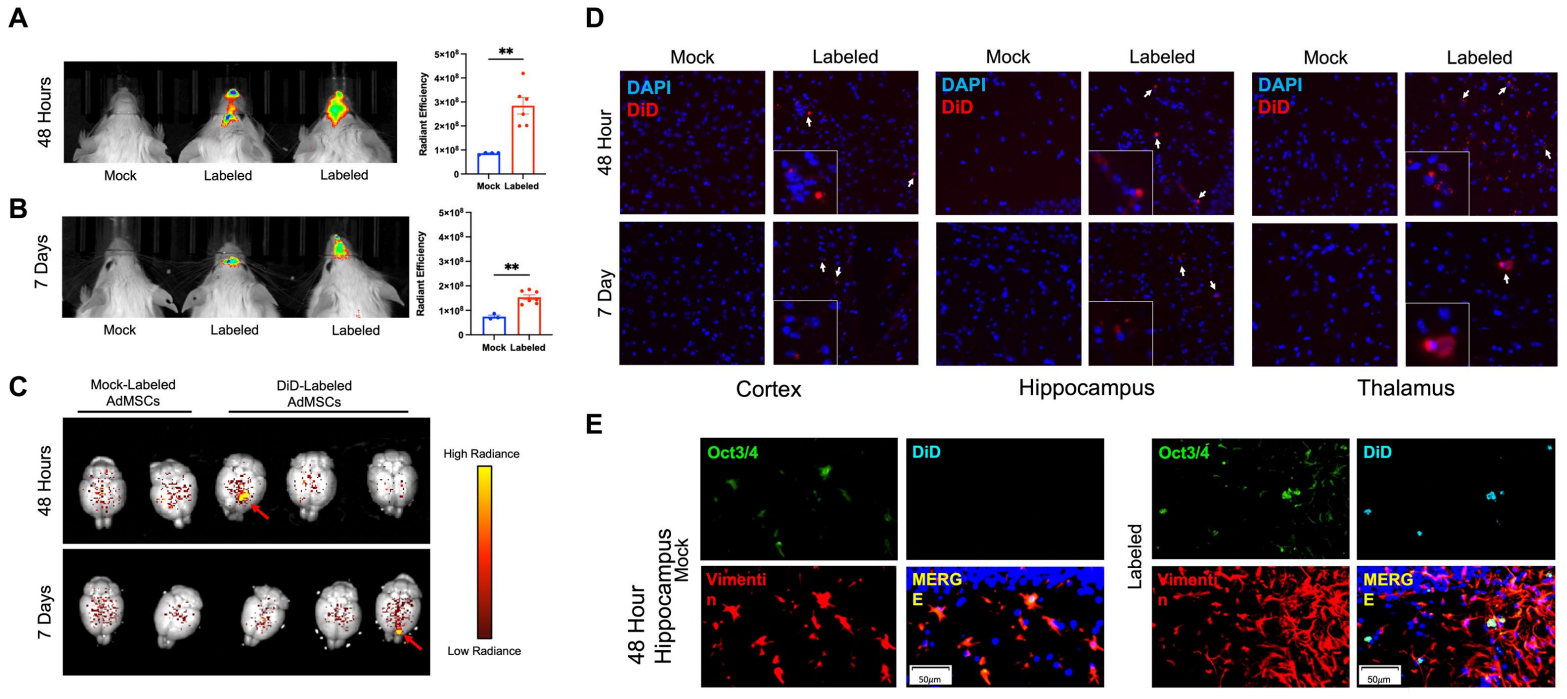
Fluorescently-labeled AdMSCs migrate deep into the olfactory system and into the brains of prion-infected mice. AdMSCs were stimulated for 24 h with TNFα, labeled with DiD lipophilic dye and delivered intranasally into mice with RML exposure at 18 weeks post infection (wpi). Control mice received unlabeled AdMSCs. Live animal imaging was performed **(A)** 48 h and **(B)** 7 days post-delivery. Both time points showed significant maximum radiance from cells in the olfactory region, determined using a *T*-test with Welch’s corrections, **p < 0.01, error bars = SEM. **(C)** At each timepoint, brains were removed and imaged. Areas of labeled cells were visible in some brains at 48 h and 7 days post-delivery (shown by red arrows). Radiance scale used for degree of fluorescent dye positivity in brains. **(D)** Representative images of DiD+ cells in the cortex, hippocampus and thalamus at 48 h and 7 days post-delivery, compared to brains from animals that received mock-labeled AdMSCs. Arrows indicate DiD+ cells. Cell nuclei are stained with DAPI. **(E)** Hippocampus of animals that received mock-labeled or DiD-labeled AdMSCs at 48 h post-delivery. Tissue was co-stained for AdMSC markers Vimentin and Oct3/4, which co-localizes with DiD staining. 20x representative images, scale bar = 50 μm.

We have shown previously that AdMSCs isolated from the abdominal adipose tissue of C57Bl/6 mice contain a heterologous population of cells with characteristics of mesenchymal stem cells. Additionally, these cells can migrate in an *in vitro* model toward the prion-infected brain (Shan et al., 2017; Hay et al., 2022). PrP KO AdMSCs were stimulated for 24 h with tumor necrosis factor alpha (TNFα) to promote migration to the prion-infected brain (Hemeda et al., 2010; Xiao et al., 2012; Yu et al., 2017) and labeled with fluorescent lipophilic dye (DiD) or mock-labeled with PBS and intranasally delivered into RML-infected CD-1 mice at 18 wpi. Live mice were imaged with the Xenogen IVIS Imaging System at 24, 48, 72 h, 7 days and 14 days post-delivery and brains were extracted and imaged at each of these timepoints. Analysis of the maximum radiance showed significantly more fluorescence signal from mice receiving the labeled AdMSCs at 24 h (*p* < 0.01; data not shown), 48 h (Figure 1A; *p* < 0.01), 72 h (*p* < 0.001; data not shown) and 7 days (Figure 1B; *p* < 0.01), but no significant difference were seen between groups at 14 days (data not shown). When brains were dissected out and imaged with the Xenogen IVIS, no significant differences in maximum radiance of whole brains were seen between mock- and labeled AdMSC-treated groups. However, specific sites of fluorescently labeled cells can be observed (Figure 1C, shown by red arrows) in some of the brains that received labeled AdMSCs. Of the brains that received labeled AdMSCs, regions of fluorescence were seen in two of four brains at 24 h, one of four brains at 48 h, and one of four brains at 7 days. No regions of fluorescence were seen at 72 h or 14 days post-delivery (data not shown), nor were they seen in mice that received mock-labeled AdMSCs.

A cohort of 12 CD-1 mice at 18wpi were given stimulated intranasally-delivered DiD- or mock-labeled PrP KO AdMSCs, following an identical protocol as above. At 48 h and 7 days post-delivery, mice were euthanized and brains were immediately frozen on dry ice. Brain sections were assessed for DiD+ cells in the cortex, hippocampus, thalamus and cerebellum. DiD+ cells were found in almost all animals in the cortex, hippocampus, and thalamus (Figure 1D), and in less than half of animals in the cerebellum at both 48 h and 7 days post-delivery (Table 2). No DiD+ cells were found in brains from mice that received mock-labeled AdMSCs. Brains from mice at 48 h post-delivery that showed significant DiD+ cells in the hippocampus were stained with the stem cell marker Oct3/4 and the structural marker Vimentin, both of which label AdMSCs (Hay et al., 2022). Note that there are no markers specific to AdMSCs that are not expressed in other cell types in the brain. Oct3/4 can also be expressed in pluripotent cells, and Vimentin is also expressed in astrocytes. All DiD+ cells analyzed were Oct3/4+ and the majority were Vimentin+ (Figure 1D). Together, these data suggest that AdMSCs delivered intranasally can migrate throughout the brain and remain there for a minimum of 7 days.

**Table 2:** DiD+ cells in brain regions after intranasal delivery.

	Cortex	Hippocampus	Thalamus	Cerebellum
Mock-Labeled	0/2	0/2	0/2	0/2
DiD – 48 h	5/5	4/5	4/5	2/5
DiD – 7 days	4/5	5/5	4/5	2/5

### 3.2. Stimulating AdMSCs with inflammatory cytokines and prion-infected brain homogenate for 24 h increases production of anti-inflammatory molecules and growth factors

**Figure 2:**
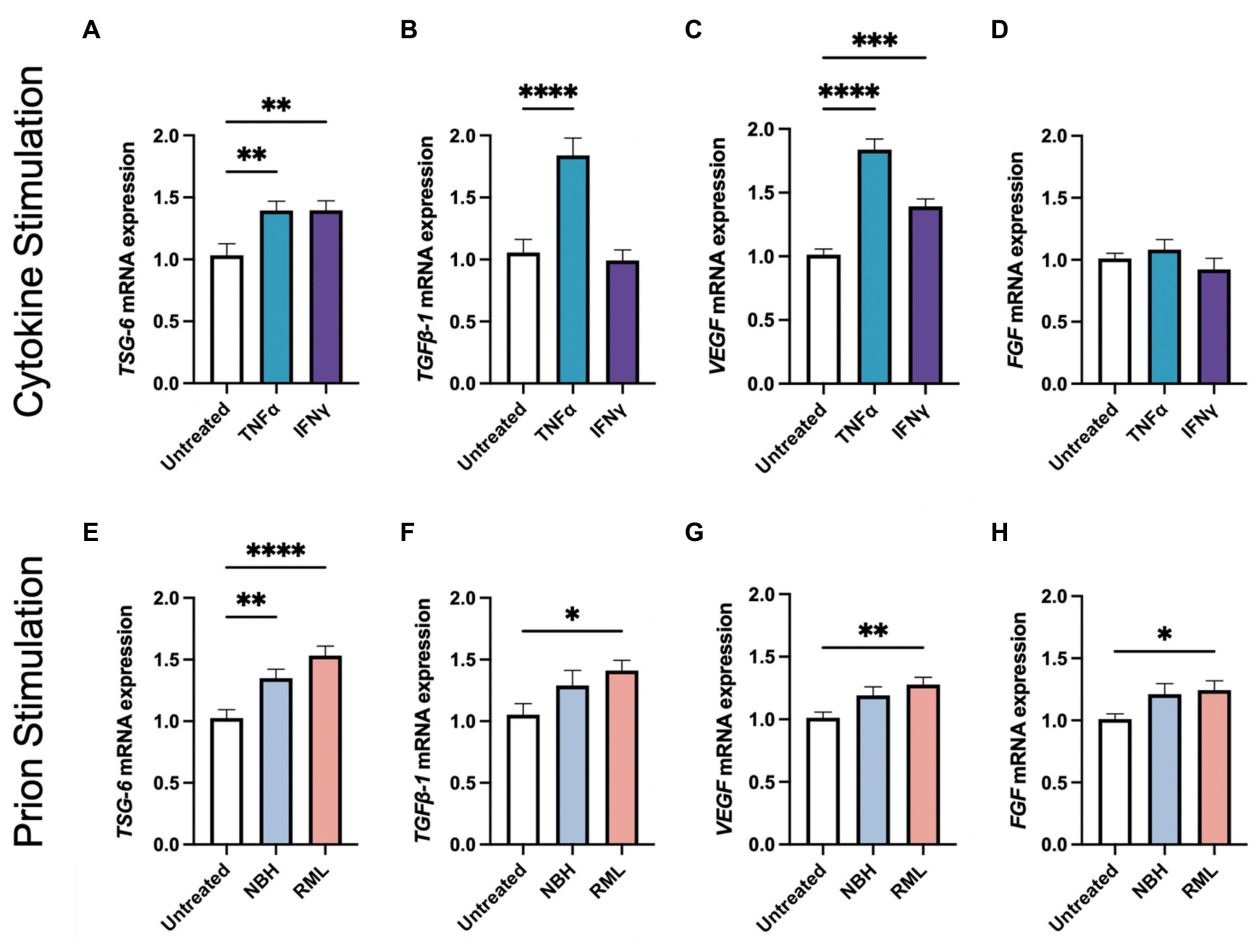
Stimulating AdMSCs with inflammatory cytokines and prion-infected brain homogenate for 24 h increases production of anti-inflammatory molecules and growth factors. AdMSCs were incubated for 24-h in media containing TNFα or IFNγ. These AdMSCs showed significant increase in mRNA expression levels for **(A)** *TSG-6*, **(B)** *TGFβ-1*, and **(C)** *VEGF*, but no changes in **(D)** *FGF.* AdMSCs were incubated for 24-h in media containing NBH or RML-infected brain homogenates. In AdMSCs exposed to RML, a significant increase was seen in **(E)** *TSG-6*, **(F)** *TGFβ-1*, **(G)** *VEGF*, and **(H)** *FGF* mRNA. One-way ANOVA with post-hoc Tukey’s test, *p < 0.05, **p < 0.01, ***p < 0.001, ****p < 0.0001, error bars = SEM. Combined data from four separate experiments, each with three technical replicates.

Our laboratory and others have demonstrated that stimulating MSCs with inflammatory molecules increases secretion of anti-inflammatory molecules and growth factors (English et al., 2007; Hemeda et al., 2010; Hay et al., 2022). Here, we demonstrate that stimulating AdMSCs for 24 h with TNFα or interferon gamma (IFNγ) induces a 36%-fold increase of TNF-stimulated gene 6 (*TSG-6*) mRNA (Figure 2A; *p* < 0.01). Stimulation with TNFα, but not IFNγ, causes a 78%-fold increase in transforming growth factor beta-1 (*TGFβ-1*) mRNA (Figure 2B; *p* < 0.0001) (Hay et al., 2022). Stimulation with TNFα induced an 83%-fold increase, and stimulation with IFNγ induced a 38%-fold increase in vascular endothelial growth factor (*VEGF*) mRNA (Figure 2C; *p* < 0.0001, *p* < 0.001, respectively), but no changes were seen in fibroblast growth factor (*FGF*) mRNA (Figure 2D).

We also demonstrate that culturing AdMSCs in media containing 0.1% Rocky Mountain Laboratories (RML) strain mouse-adapted scrapie brain homogenate elicits a similar upregulation of genes for anti-inflammatory molecules and growth factors, suggesting that AdMSCs respond to factors in the prion-infected brain, such as damage-associated molecular patterns (DAMPs) and the cytokine milieu (Hass and Otte, 2012; Carroll et al., 2018a). After 24 h in media containing normal brain homogenate (NBH) or RML, AdMSCs increased expression of *TSG-6* mRNA by 32 and 51%-fold change from baseline, respectively (Figure 2E; *p* < 0.01, *p* < 0.0001, respectively). Treatment with RML, but not NBH, increased mRNA for *TGFβ-1* by 35%-fold (Figure 2F; *p* < 0.05), *VEGF* by 27%-fold (Figure 2G; *p* < 0.01), and *FGF* by 23%-fold (Figure 2H; *p* < 0.05).

### 3.3. Prion-infected mice were treated with stimulated AdMSCs every 2 weeks

**Figure 3:**
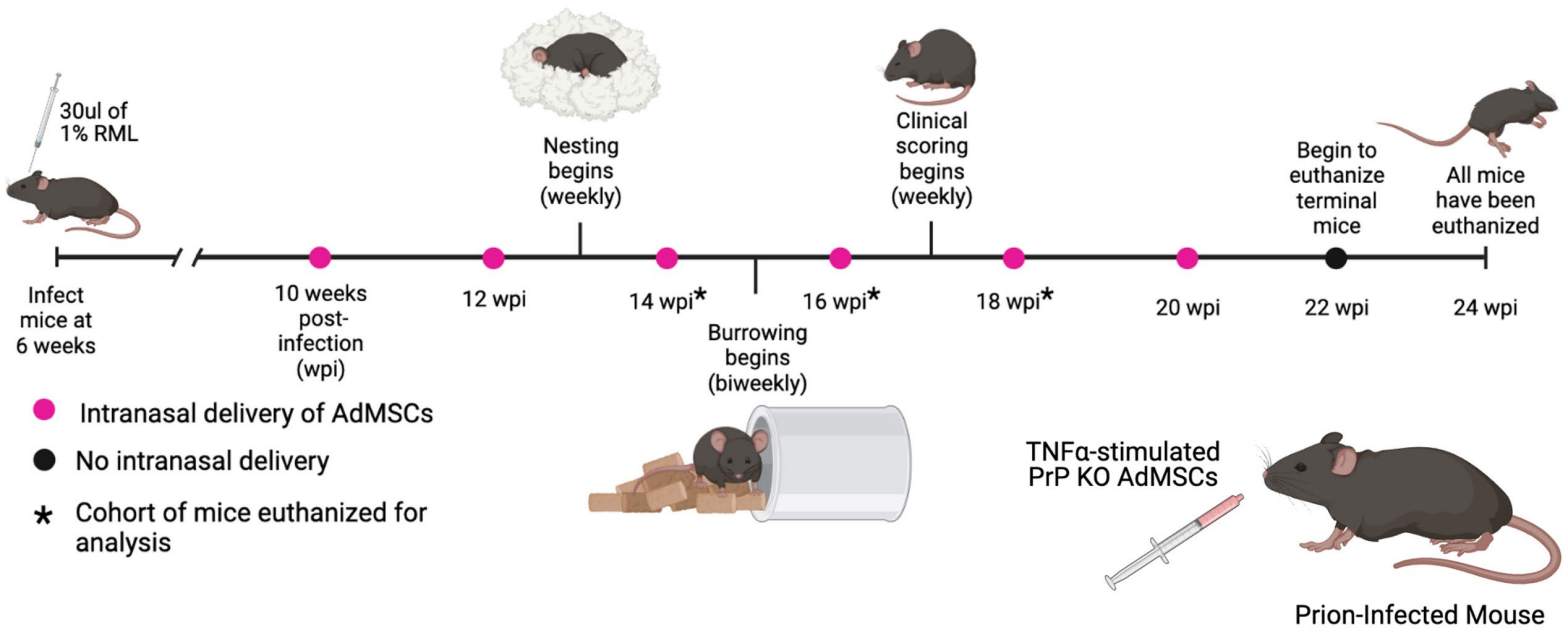
Prion-infected mice were treated with stimulated AdMSCs every 2 weeks. RML-infected mice received intranasal delivery of TNFα-stimulated PrP KO AdMSCs biweekly from 10 weeks post infection (wpi) to 20 wpi. Hippocampal-specific behavioral analyses, nesting and burrowing, were performed beginning at 13 wpi. Clinical signs were analyzed weekly beginning at 17 wpi until mice were euthanized. No changes in behavioral signs, clinical signs, or survival were observed between AdMSC-treated mice and PBS-treated controls (see Supplementary Figure S1). Graphic created with BioRender.com.

Mice received 30 μl of 1% RML mouse-adapted scrapie brain homogenate or NBH inoculated intracranially at 6 weeks of age. RML-infected mice received an intranasal delivery of 1 × 10^6^ PrP KO AdMSCs or vehicle (PBS) every 2 weeks beginning at 10 weeks post-infection (wpi) and continuing to 20 wpi (Figure 3). PrP KO AdMSCs were used instead of wild-type to eliminate any possibility of these cells becoming infected and further disseminating PrP^Sc^, as expression of PrP is critical for cells to be infectable (Bueler et al., 1993; Giese et al., 1998). Prior to each intranasal delivery, AdMSCs were incubated in media containing 10 ng/ml TNFα for 24 h. We have demonstrated here (Figure 2) and previously that this induces increased expression of anti-inflammatory molecules and growth factors. After 24 h, cells are sufficiently washed and TNFα is no longer detectable in the media (Hay et al., 2022), eliminating any concern that it may be transferred into the brain when AdMSCs are intranasally delivered.

Hippocampal-associated behavioral analysis was used to monitor early signs of disease. Nesting began at 10 wpi, but data is only shown from 13 to 22 wpi. A decline in nesting behavior was observed for the AdMSC-treated mice at 17 wpi (*p* < 0.01). At 18 wpi, both AdMSC-treated and mock-treated animals showed decline in nesting behavior (*p* < 0.001), and this was observed until mice were euthanized (*p* < 0.0001). Meanwhile, NBH mice maintained perfect nests from 18 wpi until the termination of the study at 23 weeks (Supplementary Figure S1A). Burrowing began at 13 wpi and was repeated the following 2 weeks to allow for a “training period” for the mice, before being performed every 2 weeks. Decline in burrowing was seen for mock-treated (*p* < 0.001) and AdMSC-treated mice (*p* < 0.05) beginning at 17 wpi and continuing until burrowing was discontinued after 21 wpi (*p* < 0.0001). NBH mice maintained healthy burrowing behavior through 21 weeks (Supplementary Figure S1B).

Early signs of clinical scores began for both mock- and AdMSC-treated mice at 17 wpi, but signs were not statistically significant compared to NBH mice until 21 wpi (*p* < 0.0001) (Supplementary Figure S1C). Mice were euthanized when they showed a clinical score of 10, and all mice had been euthanized before 25 wpi. No changes in survival were seen between the mock- and AdMSC-treated mice, with a mean survival of 163 days for both (Supplementary Figure S1D). NBH mice remained healthy and were euthanized after all RML-infected mice had been euthanized. To determine the effects of intranasally-delivered AdMSCs throughout disease progression, a cohort of mice was sacrificed at 14 wpi (after 3 treatments), 16 wpi (after 4 treatments), and 18 wpi (after 5 treatments). The remainder of the mice received 6 treatments total and were euthanized when they showed significant clinical signs.

### 3.4. Adipose-derived mesenchymal stromal cells treatment decreases inflammatory cytokine transcripts in the hippocampus at 16 weeks post-infection

**Figure 4:**
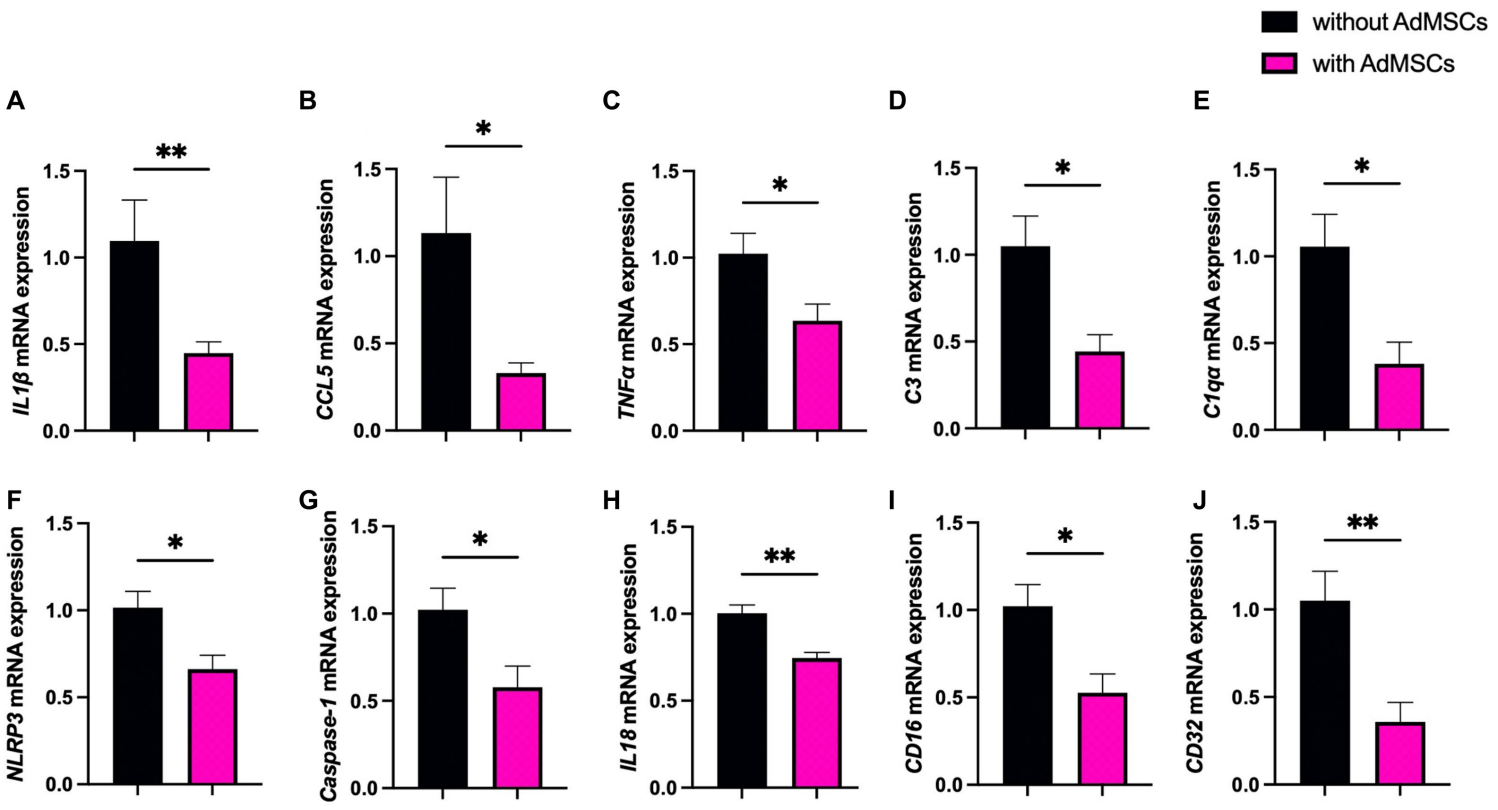
Adipose-derived mesenchymal stromal cells (AdMSC) treatment decreases inflammatory cytokine transcripts in the hippocampus at 16 weeks post infection (wpi). Hippocampal mRNA expression was decreased in animals treated with AdMSCs for **(A)** *IL1β*, **(B)** *CCL5*, **(C)** *TNFα*, **(D)** *C3*, **(E)** *C1qa*, **(F)** *NLRP3*, **(G)** *Caspase-1*, **(H)** *IL18*, **(I)** *CD16*, and **(J)** *CD32.* Hippocampi were analyzed from 10 animals, 6 AdMSC-treated and 4 PBS-treated controls. *T*-test with Welch’s corrections, *p < 0.05, **p < 0.01, error bars = SEM.

mRNA levels of genes associated with prion disease were measured in hippocampi from mice at 16 wpi and 18 wpi. At 16 wpi, AdMSC treatment led to significant decreases in inflammatory genes and markers of astrogliosis. A 68.4%-fold decrease was seen in the inflammatory molecule interleukin 1 beta (*IL1β*) (Figure 4A; *p* < 0.01), an 80.5%-fold decrease in chemokine ligand 5 (*CCL5*) (Figure 4B; *p* < 0.05), and a 38.8%-fold decrease in *TNFα* (Figure 4C; p < 0.05). AdMSC treatment caused a 60.7%-fold decrease for complement component 3 (*C3*) (Figure 4D; *p* < 0.05), and a 67.5%-fold decrease in mRNA for the complement C1q subcomponent subunit A (*C1qa*) (Figure 4E; *p* < 0.05), both of which are known contributors to the development of neurotoxic reactive astrocytes (Liddelow et al., 2017; Hartmann et al., 2019). AdMSCs induced changes in genes associated with the NLRP3 inflammasome pathway. A 35.3%-fold decrease was seen in mRNA for *NLRP3* with AdMSC treatment (Figure 4F; *p* < 0.05). mRNA for *Caspase 1*, which is recruited and activated by the NLRP3 inflammasome, decreased by 44.3%-fold upon treatment (Figure 4G; *p* < 0.05). Recruitment of Caspase-1 by NLRP3 processes inactive pro-interleukin 18 (pro-IL18) and pro-IL1β to the active forms, IL18 and IL1β (Blevins et al., 2022). As it decreased for *IL1β,* mRNA for IL18 decreased by 25.7%-fold in AdMSC treated hippocampi (Figure 4H; p < 0.01). Additionally, a 49.6%-fold decrease was shown in *CD16* (Fc gamma III receptor) (Figure 4I; *p* < 0.05) and a 69.2%-fold decrease for *CD32* (Fc gamma II receptor) (Figure 4J; *p* < 0.01), two genes associated with M1 microglia.

No changes were seen in mRNA for interleukin 6 (*IL6*), chemokine ligand 2 (*CCL2*), inducible nitric oxide synthase (*iNos*) or *NF-κB1*, or in markers of M2 microglia, arginase-1 (*Arg-1*) or insulin-like growth factor (*Igf-1*) (Supplementary Figure S2). Additionally, no significant changes were seen in any of the genes analyzed in the hippocampi of mice at 18 wpi (Supplementary Figure S3).

### 3.5. Less vacuolization throughout the brains of AdMSC-treated mice at 16 wpi

**Figure 5:**
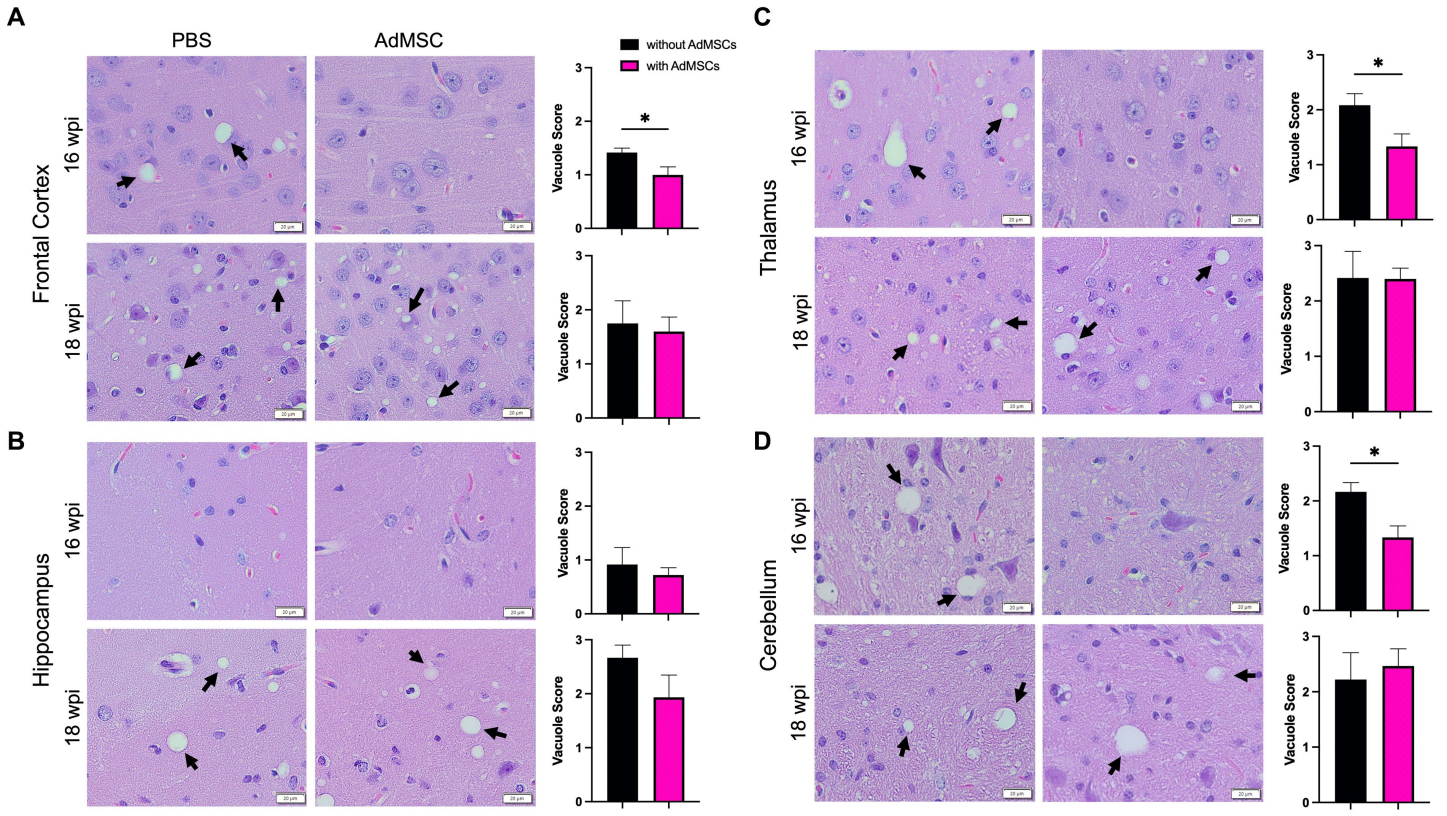
Less vacuolization throughout the brain of AdMSC-treated mice at 16 weeks post infection (wpi). Vacuoles in the **(A)** frontal cortex, **(B)** hippocampus, **(C)** thalamus, and **(D)** cerebellum were scored on a scale of 0–5 based on size and number. An average of three scores for each brain region was calculated for each animal. Sixteen wpi AdMSC-treated mice had decreased vacuoles in all brain regions except for the hippocampus. No differences in vacuolization were detected between treatment groups for 18 wpi animals. H&E stained brains were analyzed from 10 animals per timepoint, 6 AdMSC-treated and 4 PBS-treated controls. *T*-test with Welch’s corrections, *p < 0.05, error bars = SEM. 40x representative images, scale bar = 20 μm.

Fixed tissue was stained with hematoxylin and eosin and images of the frontal cortex, hippocampus, thalamus and cerebellum were taken. Three pathologists who were blinded to the treatment groups scored each brain region from 0 (no vacuolization) to 5 (severe vacuolization) based on size and number of vacuoles (Fraser and Dickinson, 1968; Scott et al., 1997; Sun et al., 2023). Significant vacuolization can be seen in the brains of terminal prion-infected mice in these brain regions, compared to healthy mice from age-matched mice that received NBH brain inoculum (Supplementary Figure S4; *p* < 0.0001). At 16 wpi, brains with AdMSC treatment showed significantly less vacuolization in the frontal cortex (PBS mean = 1.41, SD = 0.167; AdMSC mean = 1.00, SD = 0.365), thalamus (PBS mean = 2.08, SD = 0.419; AdMSC mean = 1.33, SD = 0.558) and cerebellum (PBS mean = 2.17, SD = 0.333; AdMSC mean = 1.33, SD = 0.471) (Figures 5A–D; *p* < 0.05). Interestingly, no differences were seen in vacuole severity in the hippocampus at this timepoint (PBS mean = 0.917, SD = 0.631; AdMSC mean = 0.722, SD = 0.328). No differences were seen in vacuolization at 18 wpi (Figures 5A–D), nor were there significant changes in vacuoles at 14wpi, prior to development of clinical signs or at terminal disease (data not shown).

### 3.6. Adipose-derived mesenchymal stromal cells treatment does not induce detectable changes in PrP^Sc^

**Figure 6:**
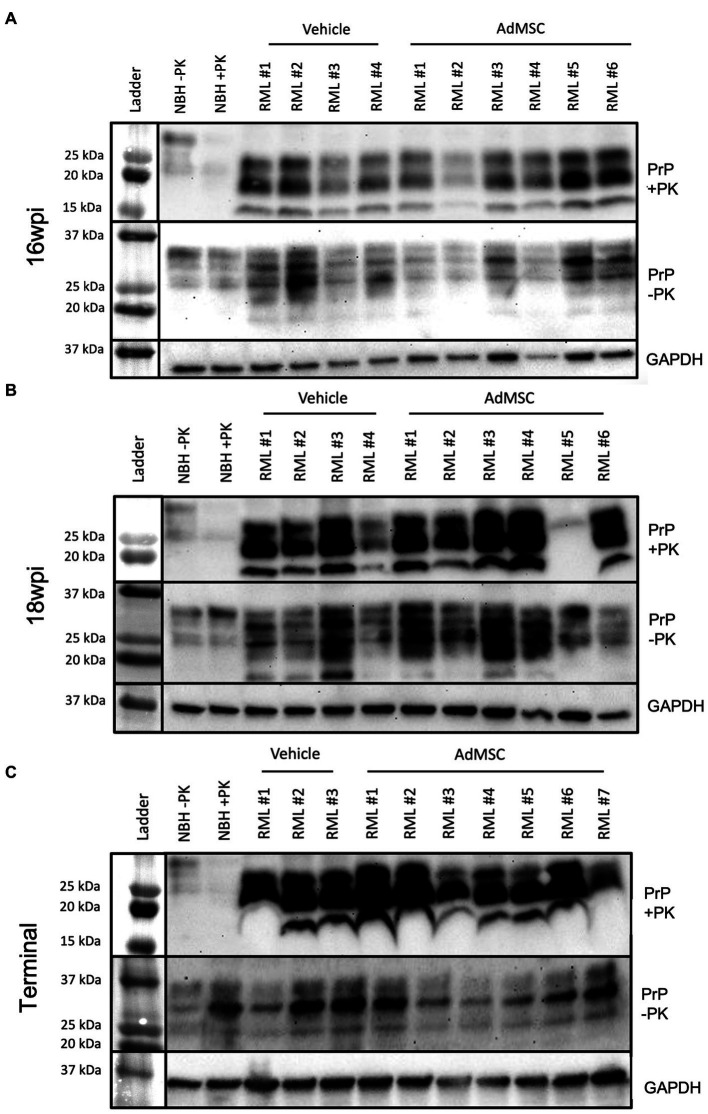
Adipose-derived mesenchymal stromal cells treatment does not induce detectable changes in PrP^Sc^. Western blots were used to compare both PrP^Sc^ (PK-resistant PrP) and total PrP (not PK-treated) between vehicle and AdMSC-treated mice at **(A)** 16 wpi, **(B)** 18 wpi, and **(C)** terminal stages of disease with no detectable differences between treatments (see Supplementary Figure S7 for quantification). GAPDH is used as loading control. Brain homogenates were analyzed from 10 animals per timepoint, 6 AdMSC-treated and 4 PBS-treated controls (for terminal mice, 7 AdMSC-treated and 3 PBS-treated controls).

Brains from mice at 16 wpi, 18 wpi, and terminal stage disease were homogenized and analyzed *via* western blot to detect both total PrP and the disease-associated proteinase-K (PK) resistant infectious PrP (PrP^Sc^). Equivalent amounts of protein were loaded and band intensity was analyzed in ImageJ. A Rout’s test was used to identify outliers. One animal in the 18 wpi cohort (labeled RML #5) showed significantly lower PrP^Sc^ (but comparable PrP^C^) to the rest of its cohort. This animal likely did not receive a full dose of RML brain homogenate when inoculated, and was therefore removed from all histological data analyses. Band intensity from all other brain samples was compared between treated and untreated groups (Supplementary Figure S7). Normal brain homogenate (NBH) with and without PK was used as a negative control. AdMSC treatment did not induce changes in either total PrP or PrP^Sc^ in 16 wpi (Figure 6A), 18 wpi (Figure 6B) or terminal brain homogenates (Figure 6C).

### 3.7. Death of hippocampal neurons is not prevented by AdMSC treatment

**Figure 7:**
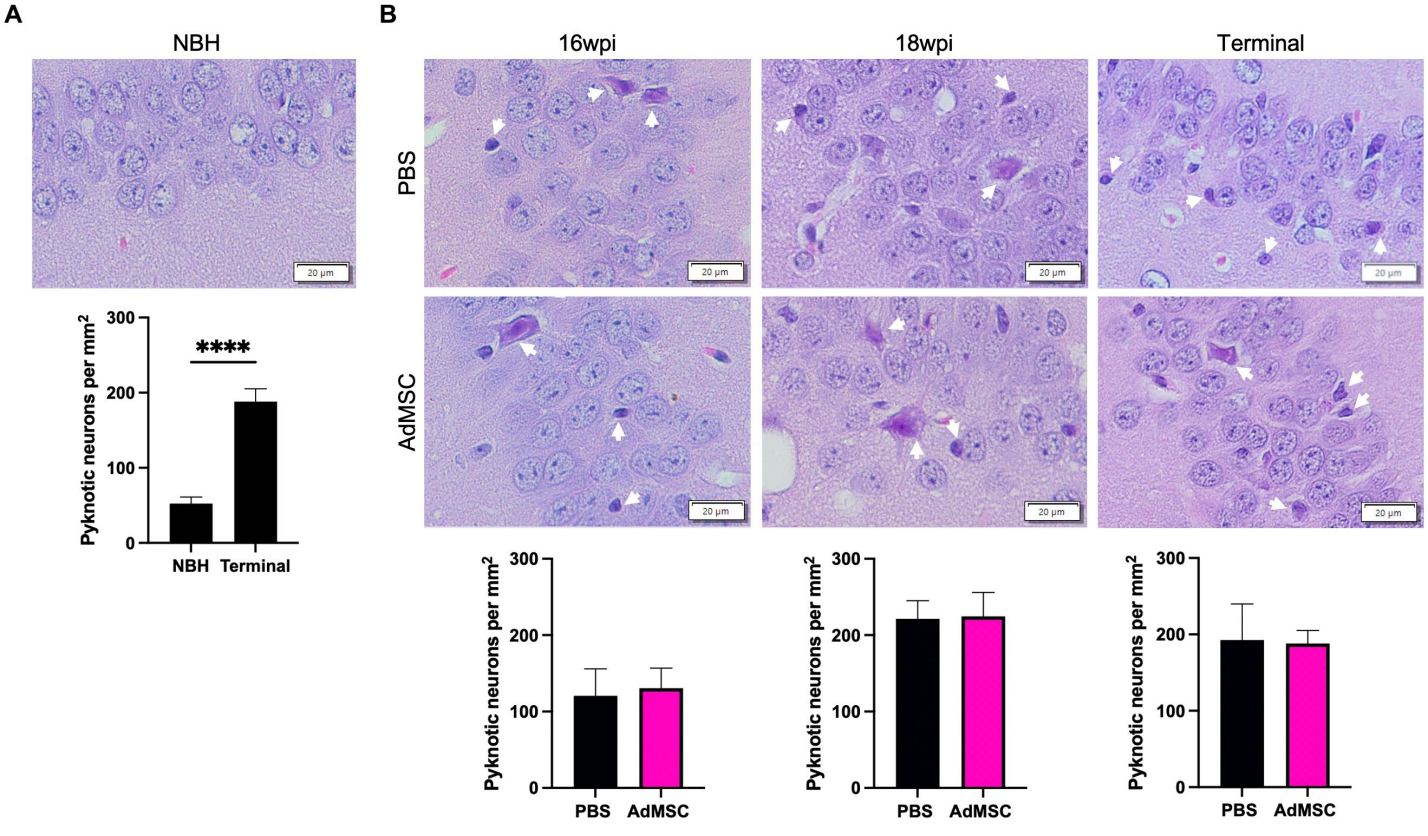
Death of hippocampal neurons is not prevented by AdMSC treatment. Swollen and pyknotic neurons in the CA1 region of the hippocampus were compared. **(A)** Significantly more pyknotic neurons were seen in terminally-infected mice when compared to age-matched NBH mice. **(B)** No changes were seen between AdMSC-treated and control mice at 16 wpi, 18 wpi or terminal stages of infection. Hippocampi were analyzed from 10 animals per timepoint, 6 AdMSC-treated and 4 PBS-treated controls. *T*-test with Welch’s corrections, ****p < 0.0001, error bars = SEM. IHC: 20x, IF: 40x representative images, scale bar = 20 μm.

Pyknotic neurons were assessed in tissue stained with hematoxylin and eosin. Specifically, both swollen/apoptotic and shrunken neurons were manually counted in the CA1 region of the hippocampus (Petito et al., 1997; Majer et al., 2012; Moreno et al., 2012). Significantly more neuronal death was seen in terminal mice regardless of treatment compared to NBH mice (Figure 7A; *p* < 0.0001). AdMSC treatment did not induce changes in the number of pyknotic neurons at 16 wpi, 18 wpi or terminal stages of disease (Figure 7B).

### 3.8. Adipose-derived mesenchymal stromal cells treatment promotes ramified microglia in the hippocampus

**Figure 8:**
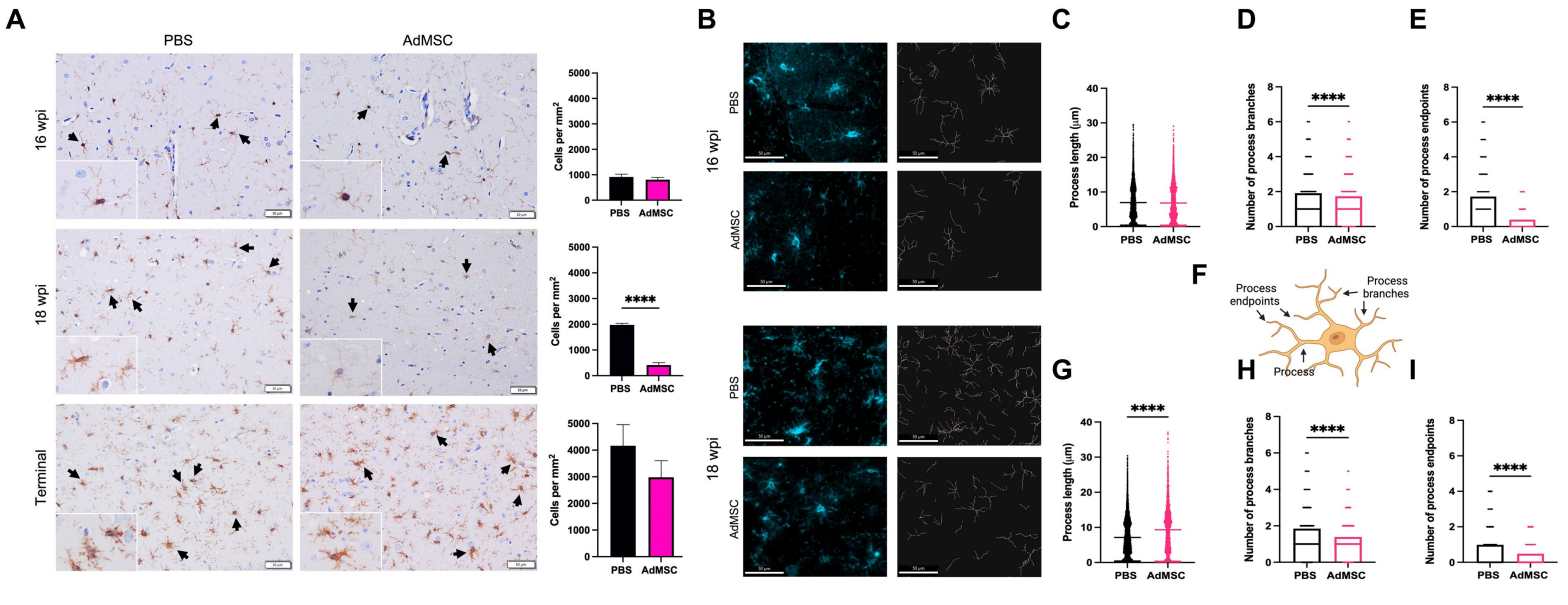
Adipose-derived mesenchymal stromal cells treatment promotes ramified microglia in the hippocampus. **(A)** Counts of Iba1+ cells were performed in the hippocampus. **(B)** Skeletons of Iba1+ hippocampal cells were analyzed with IMARIS for mice at 16 weeks post infection (wpi) and 18 wpi. Quantification of process length **(C)** in PBS and AdMSC treated mice. AdMSC-treated animals at 16 wpi had no change in process length, but **(D)** fewer process branches and **(E)** fewer process endpoints. **(F)** Illustration depicting processes, process branches, and process endpoints in microglial cell. AdMSC-treated animals at 18 wpi had **(G)** longer processes, and **(H)** fewer branches and **(I)** fewer endpoints. Hippocampi were analyzed from 10 animals per timepoint, 6 AdMSC-treated, and 4 PBS-treated controls. *T*-test with Welch’s corrections, ****p < 0.0001, error bars = SEM. IHC: 20x, IF: 40x representative images, scale bar = 50 μm. Graphic created with BioRender.com.

The marker Iba1 was used to stain and count microglia throughout the brain. Surprisingly, no changes were seen in microglia number at 16 wpi in treated mice. At this time point microglia remained sparse in the hippocampus. At 18 wpi, however, AdMSC-treated mice showed a significant decrease in microglia number in the hippocampus compared to PBS-treated controls (Figure 8A; *p* < 0.0001). No differences were seen between treated and untreated mice in number of microglia in terminal mice, or between treatment groups in the frontal cortex, thalamus or cerebellum (representative images of thalamic microglia are available in Supplementary Figure S5).

Morphology of hippocampal microglia was analyzed using IMARIS software (Figure 8B) to identify process length, number of process branches, and number of process endpoints for each cell (see Figure 8F for examples). Differences in microglia morphology were less visibly apparent in mice at 16 wpi. However, quantification with IMARIS demonstrated that although process length was equivalent between AdMSC-treated animals and controls, the number of process branches was significantly lower in AdMSC-treated microglia (PBS mean = 1.91, AdMSC mean = 1.73, *p* < 0.0001). This was also associated with fewer process endpoints (PBS mean = 1.72, AdMSC mean = 0.39, *p* < 0.0001). At 18 wpi, microglia from AdMSC-treated mice had longer processes (PBS mean = 7.16 μm, AdMSC mean = 9.33 μm, p < 0.0001), fewer process branches (PBS mean = 1.85, AdMSC mean = 1.39, *p* < 0.0001), and fewer process endpoints (PBS mean = 0.98, AdMSC mean = 0.48, *p* < 0.0001) (Figures 8C–I). Together, these data suggest that AdMSCs are inducing a phenotypic change in hippocampal microglia, promoting ramified, homeostatic microglia as opposed to activated, amoeboid microglia (Soulet and Rivest, 2008).

### 3.9. Adipose-derived mesenchymal stromal cells treatment decreases reactive astrocytes in the hippocampus

**Figure 9:**
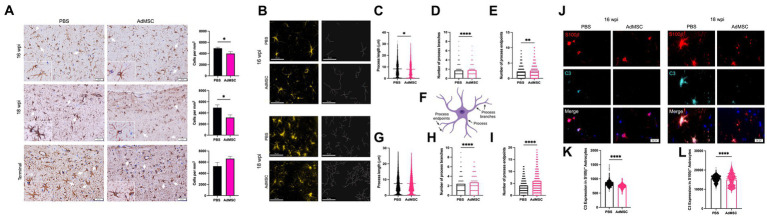
Adipose-derived mesenchymal stromal cells treatment decreases reactive astrocytes in the hippocampus. **(A)** Counts of GFAP+ cells were performed in the hippocampus. **(B)** Skeletons of GFAP+ hippocampal cells were analyzed with IMARIS for mice at 16 weeks post infection (wpi) and 18 wpi. AdMSC-treated animals at 16 wpi had **(C)** slightly shorter processes, and **(D)** more process branches and **(E)** more process endpoints. **(F)** Illustration depicting processes, process branches, and process endpoints in an astrocyte. AdMSC-treated animals at 18 wpi had **(G)** no change in process length, but **(H)** more branches and **(I)** more endpoints. Expression of C3 in S100β + hippocampal astrocytes was analyzed for mice at **(J)** 16 and 18 wpi with significantly less C3 expression seen in S100β + astrocytes from mice treated with AdMSCs for both **(K)** 16 wpi and **(L)** 18 wpi hippocampi (arbitrary units). Hippocampi were analyzed from 10 animals per timepoint, 6 AdMSC-treated and 4 PBS-treated controls. *T*-test with Welch’s corrections, *p < 0.05, **p < 0.01, ****p < 0.0001, error bars = SEM. Representative images for IHC are at 20x, scale bar is 50 μm. Representative images for IF are at 40x, scale bar is 50 μm for skeletonization and 50 μm for C3 expression. Graphic created with BioRender.com.

The pan-astrocyte marker glial fibrillary acidic protein (GFAP) was used to stain and count astrocytes throughout the brain. In mice that received intranasal delivery of AdMSCs, significantly fewer astrocytes were seen in the hippocampus at both 16 and 18 wpi (Figure 9A; *p* < 0.05), but no differences were seen in terminal mice. No differences were seen between treated and untreated mice in number of GFAP+ astrocytes in the frontal cortex, thalamus or cerebellum (representative images of thalamic astrocytes are available in Supplementary Figure S6).

Morphology of hippocampal GFAP+ astrocytes was analyzed using IMARIS software (Figure 9B) to identify process length, number of process branches, and number of process endpoints for each cell (see Figure 9F for examples). At 16 wpi, astrocytes from AdMSC-treated mice had slightly shorter processes (PBS mean = 8.30 μm, AdMSC mean = 7.95 μm, *p* < 0.05), more process branches (PBS mean = 1.724, AdMSC mean = 1.793, *p* < 0.0001), and more process endpoints (PBS mean = 2.276, AdMSC mean = 2.565, *p* < 0.01). At 18 wpi there was no difference in process length between treatment groups. AdMSC-treated mice had significantly more astrocyte process branches (PBS mean = 2.42, AdMSC mean = 2.71, *p* < 0.0001), and process endpoints (PBS mean = 4.05, AdMSC mean = 5.99, *p* < 0.0001) (Figures 9C–I). Together, these data suggest that AdMSCs are inducing a phenotypic change in hippocampal astrocytes, promoting a homeostatic, neuroprotective phenotype.

Colocalization of S100β and the complement protein C3 is a marker for a subset of reactive neurotoxic astrocytes in a variety of neurodegenerative diseases (Liddelow et al., 2017). The mean fluorescence intensity of C3 in S100β + cells was quantified in the hippocampus of mice at 16 and 18 wpi. At both timepoints, significantly less C3 was identified in animals that had been treated with AdMSCs (Figures 9J–L; *p* < 0.0001), suggesting that AdMSCs are decreasing the number of reactive astrocytes in the hippocampus.

## 4. Discussion

Mesenchymal stromal cells (MSCs) can migrate toward prion-infected brain homogenate *in vitro* (Shan et al., 2017; Hay et al., 2022). In this study, the therapeutic capacity of intranasal delivery of AdMSCs was investigated in mice inoculated with mouse-adapted scrapie prions. We labeled AdMSCs with a fluorescent, lipophilic dye called DiD and intranasally delivered them into mice with RML scrapie at 18 wpi. We removed the brains from a cohort of mice at 48 h and 7 days and found areas of DiD+ cells in approximately one out of four brains (Figure 1). Assessment of sagittal brain sections identified DiD+ cells in the cortex, hippocampus, thalamus and cerebellum of animals at both 48 h and 7 days post-delivery, suggesting that AdMSCs delivered intranasally can migrate throughout the brain and remain for at least 7 days (Table 2). DiD+ cells assessed in the hippocampus of mice at 48 h post-delivery were also positive for Oct3/4 and Vimentin, two markers of AdMSCs (Hay et al., 2022).

MSCs are known to respond to an inflammatory environment by producing anti-inflammatory and protective factors. To mimic the inflammatory environment of the prion-infected brain, AdMSCs were incubated in media containing either inflammatory cytokines TNFα or IFNγ, or brain homogenate from terminally-infected mice with RML-scrapie for 24 h. Both cytokines and RML brain homogenates resulted in an upregulation in *TSG-6, TGFβ1, VEGF,* and *FGF* (Figure 2). TSG-6 is an immunomodulator produced by MSCs and has been linked to decreasing NF-κB and other inflammatory signaling in glia, and reprogramming M1 microglia to an M2 phenotype (Liu et al., 2019; Yang et al., 2020; Tang et al., 2021). TGFβ1 promotes neurogenesis by promoting quiescent microglia in mouse models of prion disease (Boche et al., 2006; De Lucia et al., 2016). VEGF promotes angiogenesis and tissue repair and decreases inflammatory cytokines in the brain (Xu et al., 2017), and FGF downregulates NF-κB signaling in microglia and decreases astrogliosis in models of traumatic brain injury (Dordoe et al., 2022; Qin et al., 2022). Together, this data suggests that stimulation of AdMSCs with inflammatory cytokines such as TNFα increases their production of protective molecules. The exact factors that AdMSCs are responding to in crude brain homogenate from RML-infected animals is unclear, although we hypothesize it is a combination of DAMPs and inflammatory cytokines (English et al., 2007; Hemeda et al., 2010; Hass and Otte, 2012; Carroll et al., 2018a). Our data suggests that, when delivered intranasally, stimulated AdMSCs will migrate to the brain of prion-infected mice and secrete protective, anti-inflammatory factors. They can remain in the brain for at least 7 days, and during this time will continue producing protective factors in response to the cytokine milieu in the prion-infected brain.

Intranasal delivery of AdMSCs was performed every other week on RML-infected mice, beginning at 10 wpi and ending at 20 wpi (Figure 3). Prior to delivery, AdMSCs were stimulated for 24 h with TNFα to increase production of anti-inflammatory genes (Hay et al., 2022), and to promote homing to inflammatory tissue (Hemeda et al., 2010; Xiao et al., 2012; Yu et al., 2017). mRNA analysis of the hippocampus in mice at 16 and 18 wpi was performed (after 4 or 5 AdMSC deliveries, respectively). Mice at 16 wpi that received AdMSCs demonstrated a significant decrease in the inflammatory cytokines *IL1β, CCL5,* and *TNFα,* as well in the complement proteins *C3* and *C1qa* which are associated with reactive astrocytes and phagocytic microglia (Hong et al., 2016; Liddelow et al., 2017; Hartmann et al., 2019). AdMSC treatment decreased mRNA transcript levels for genes associated with the NLRP3 inflammasome, *NLRP3, Caspase-1,* and *IL-18*, which have been implemented in *in vitro* models of prion infection (Shi et al., 2012; Lai et al., 2018). *CD16* and *CD32,* two markers of M1 microglia, were also decreased at 16 wpi in AdMSC-treated hippocampi, however no changes were seen in markers for M2 microglia (Figure 4). Interestingly, no significant changes were seen in mRNA transcript levels in the brains of mice at 18 wpi, despite cellular changes being apparent at this timepoint (Figures 8, 9), suggesting that AdMSCs only regulated mRNA expression for these genes early in disease (Supplementary Figure S2).

Astrogliosis, neuronal loss, and the development of spongiform tissue throughout the brain are hallmarks of prion diseases, and though poorly understood, the development of vacuoles is indirectly attributed to neuronal death (Sigurdson et al., 2019). Both size and quantity of vacuoles were assessed to score spongiform development in the frontal cortex, hippocampus, thalamus and cerebellum. Interestingly, a significant decrease in vacuolization was only seen in the frontal cortex, thalamus and cerebellum of AdMSC-treated mice at 16 wpi, not in the hippocampus (Figure 5). Changes in mRNA expression and astrocyte and microglia morphology (described below) were seen in the hippocampus at this time point, suggesting that inflammation and astrogliosis may be separate from the development of vacuoles. It was recently reported that prion-induced depletion of the phosphoinositide kinase PIKfyve causes vacuolation in prion-infected cells, neurons and brains (Lakkaraju et al., 2021). Heterogeneous PIKfyve depletion during prion infection and AdMSC treatment may uncouple these prion disease processes, or the timing of them.

The number of microglia was assessed throughout the brain using Iba1 staining. Significantly fewer microglia were seen in the hippocampus of mice treated with AdMSCs at 18 wpi (Figure 8). The visual appearance of these microglia suggests a homeostatic quiescent phenotype (Nimmerjahn et al., 2005), with longer and fewer processes, whereas the microglia of vehicle-treated mice have more and shorter processes and appear amoeboid, indicative of an activated phenotype (Dihne et al., 2001; Jurga et al., 2020). Skeletonization of Iba1+ microglia in the hippocampus demonstrated a significant decrease in process branch number and process endpoint number with AdMSC treatment at both 16 and 18 wpi, in addition to significantly longer processes in microglia at 18 wpi (Figure 8). Together, these data suggest that intranasally-delivered AdMSCs polarize microglia toward a ramified, neuroprotective phenotype. The ability of AdMSCs to reprogram microglia has been demonstrated by our lab *in vitro* using BV2 microglia (Hay et al., 2022). Additionally, it has been shown with intracranially-delivered bone marrow-derived or compact bone-derived MSCs in the context of prion disease and other neuroinflammatory diseases (Song et al., 2009; Shan et al., 2017; Liu et al., 2019). Interestingly, a decrease in markers of M1 microglia, *CD16* and CD32, was only observed in mice treated with AdMSCs at 16 wpi (Figure 4), but not at 18wpi (Supplementary Figure S3), and no changes are seen in *Arg-1* or *Igf-1* mRNA (Supplementary Figure S4), two markers of M2 microglia. These observations suggest that although AdMSCs promote polarization of microglia toward a homeostatic state, it is not characteristic of the classic M2 phenotype. Further investigation is required to fully characterize these microglia.

The pan-astrocyte marker GFAP was used to quantify astrocyte numbers throughout the brain. A decrease in GFAP+ astrocytes was seen in the hippocampus of AdMSC-treated mice at 16 and 18 wpi (Figure 9). At both timepoints, GFAP+ astrocytes from AdMSC-treated animals appear star-like with more branches, indicative of homeostatic astrocytes. Astrocytes from vehicle controls appear amoeboid, suggesting that they are activated, characteristic of hippocampal astrocytes in prion-infected mice (Makarava et al., 2019). Skeletonization analyses of these cells revealed significantly more process branches and terminal endpoints in those from AdMSC-treated animals, indicative of a less reactive phenotype. AdMSC treatment decreased C3 expression in S100β + astrocytes, further suggesting that AdMSCs limit astrogliosis in the hippocampus. Neurotoxic, formerly called A1, astrocytes upregulate C3 in prion diseases and other neuroinflammatory and neurodegenerative diseases (Liddelow et al., 2017; Hartmann et al., 2019).

Early stages of prion pathogenesis include astrogliosis and the activation of microglia and associated inflammation, followed by detectable accumulation of PrP^Sc^, synaptic dysfunction and neuronal cell death (Collinge and Clarke, 2007; Moreno et al., 2012; Sandberg et al., 2014; Smith et al., 2020). The neurotoxic agent in prion disease has not been fully elucidated. PrP^Sc^ itself does not kill neurons in standard 2D cell culture models, suggesting that involvement of other cell types is critical for neurotoxicity (Groveman et al., 2021). *In vivo* studies that modulate expression and phenotypes of microglia and astrocytes demonstrate that these cells play a dual role, contributing to both attenuation of disease as well as neurotoxicity (Gomez-Nicola et al., 2013; Carroll et al., 2018b; Hartmann et al., 2019; Bradford et al., 2022). We have demonstrated that AdMSCs can modulate transcript expression of markers of astrogliosis and inflammation early in prion disease, dampen activation of microglia and alleviate neurotoxic morphology in astrocytes in the hippocampus of prion-infected mice. However, we found no evidence that AdMSCs prevent neuronal loss in the hippocampus (Figure 7), which was consistent with behavioral assessments, clinical scores and survival (Supplementary Figure S1).

Our *in vitro* analysis of AdMSCs suggest that they decrease astrogliosis but have no effect on PrP^Sc^ accumulation in glial cells (Hay et al., 2022). Analysis of brain homogenate *via* western blot demonstrated similar findings, as there were no changes in either PK-resistant PrP^Sc^ or total PrP (Figure 6). These findings suggest that AdMSCs have no effects on PrP^Sc^ accumulation when delivered intranasally. The requirement of PrP^C^ expression in neurons for neuronal death to occur (Giese et al., 1998; Mallucci et al., 2003; Lakkaraju et al., 2022) suggests that targeting PrP^Sc^ may be critical to prevent neurodegeneration. Although AdMSCs demonstrated effective attenuation of reactive astrocytes and reprogramming of microglia, the burden of prion aggregation was ultimately overpowering.

Bi-weekly administration of AdMSCs beginning at 10 weeks after prion infection may not be frequent or early enough to halt irreversible neuronal loss. We showed that intranasally-delivered AdMSCs are detectable in the brain using our imaging methods at 7 days, but not after 14 days (Figure 1). Prion-induced neurodegeneration is rapid, with animal models succumbing to disease within weeks after development of behavioral and clinical signs. Moreover, once animals reached late stages of disease, undergoing anesthesia required for intranasal delivery became too stressful and potentially lethal, so treatments were stopped after 20 wpi. Effective AdMSC therapy for prion disease may require prophylactic and more frequent treatment to prevent the astrocyte induced inflammation that precedes, and may trigger, later pathologic processes and signs of prion diseases. Intranasally-delivered AdMSCs do not decrease PrP^Sc^ accumulation directly or indirectly, nor do they prevent hippocampal neuronal loss or vacuole development at late stages of disease. Together, these findings suggest that intranasal delivery of AdMSCs alone may not be an effective therapeutic to prevent prion-induced neurodegeneration, particularly in this aggressive model of intracranially-inoculated RML-scrapie. Studies are underway to assess the benefits of AdMSCs delivered intranasally or intracranially prior to disease onset in a mouse model of a human genetic prion disease.

We demonstrate the ability of AdMSCs to modulate astrogliosis through the relatively non-specific but therapeutically relevant delivery method of intranasal injection. The remarkable ability of these cells to migrate to the prion-infected brain, produce cytokines and growth factors, and to attenuate neurotoxic astrogliosis and restore microglia to a homeostatic, ramified phenotype, suggests an appeal for refinement of this therapeutic approach. A current avenue of interest is the utilization of extracellular vesicles (EVs) derived from mesenchymal stromal cells (Liu et al., 2020; Peng et al., 2022). EVs are widely considered to be responsible for trafficking and delivering MSC-derived paracrine modulators. Their compact size allows them to more effectively cross the blood–brain barrier. EVs have a long half-life, and a cell-free therapy poses no threat of tumor development. MSC-derived EVs naturally contain unique arrays of neuromodulators, immune mediators, growth factors, and microRNAs that have been shown to inhibit aggregation of proteins such as amyloid-β and α-synuclein. Moreover, purified EVs can be modulated to contain specific cargo loads (Rezaie et al., 2022; Yari et al., 2022). Further investigation is required to identify specific MSC-derived molecules that mediate astrogliosis, and to optimize stimulation or “priming” of MSCs to promote optimal EV conditions to combat neurodegenerative diseases such as prion disease. The ideal treatment for prion disease would include a regulator of astrogliosis and inflammation, in combination with a therapeutic that can ameliorate PrP^Sc^ accumulation and restore neuronal health.

## Data availability statement

The raw data supporting the conclusions of this article will be made available by the authors, without undue reservation.

## Author contributions

AJDH, MZ, and JM conceived and designed the project. AJDH, AL, GM, ADH, SR, EG, CS, and VG performed the experiments. AJDH, AL, and GM contributed to data analysis. AJDH wrote the manuscript. All authors reviewed manuscript and AJDH, MZ and JAM edited manuscript.

## Funding

The Boettcher Webb-Waring Biomedical Research Award, the CSU College of Veterinary Medicine and Biomedical Sciences Murphy Turner Fund, and CSU College of Veterinary Medicine and Biomedical Sciences College Research Council.

## Conflict of interest

The authors declare that the research was conducted in the absence of any commercial or financial relationships that could be construed as a potential conflict of interest.

## Publisher’s note

All claims expressed in this article are solely those of the authors and do not necessarily represent those of their affiliated organizations, or those of the publisher, the editors and the reviewers. Any product that may be evaluated in this article, or claim that may be made by its manufacturer, is not guaranteed or endorsed by the publisher.
