# Supplementary material for: Intranasally delivered mesenchymal stromal cells decrease glial inflammation early in prion disease
Source: Front Neurosci. 2023 May 12;17:1158408. doi: 10.3389/fnins.2023.1158408 (PMC10213210; doi:10.3389/fnins.2023.1158408)
Supplement: Supplementary file 1 [file Data_Sheet_1.docx]

**Supplemental Figures**


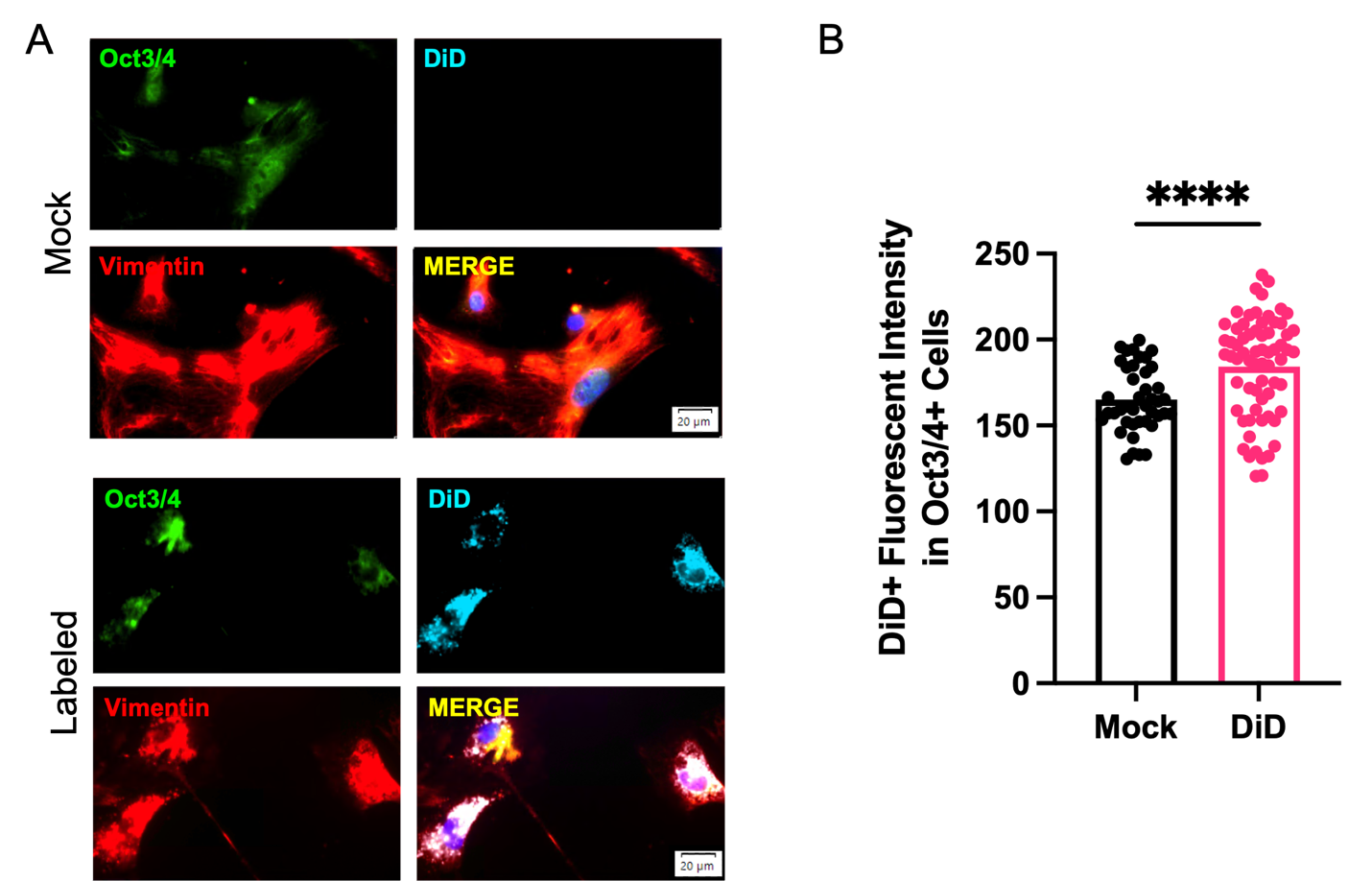


## Supplemental Figure 1. AdMSCs can be labeled with DiD and stain positive for Oct3/4 and Vimentin in vitro and in vivo. (A) AdMSCs were labeled with DiD or mock (PBS) and incubated on chamber slides for 48 hours prior to staining with Oct3/4 and vimentin. (B) DiD fluorescence was measured in Oct3/4+ cells in the hippocampus and thalamus of mice that received intranasally delivered mock- or DiD-labeled AdMSCs (arbitrary units). T-test with Welch’s corrections, ****p<0.0001, error bars = SEM.

##
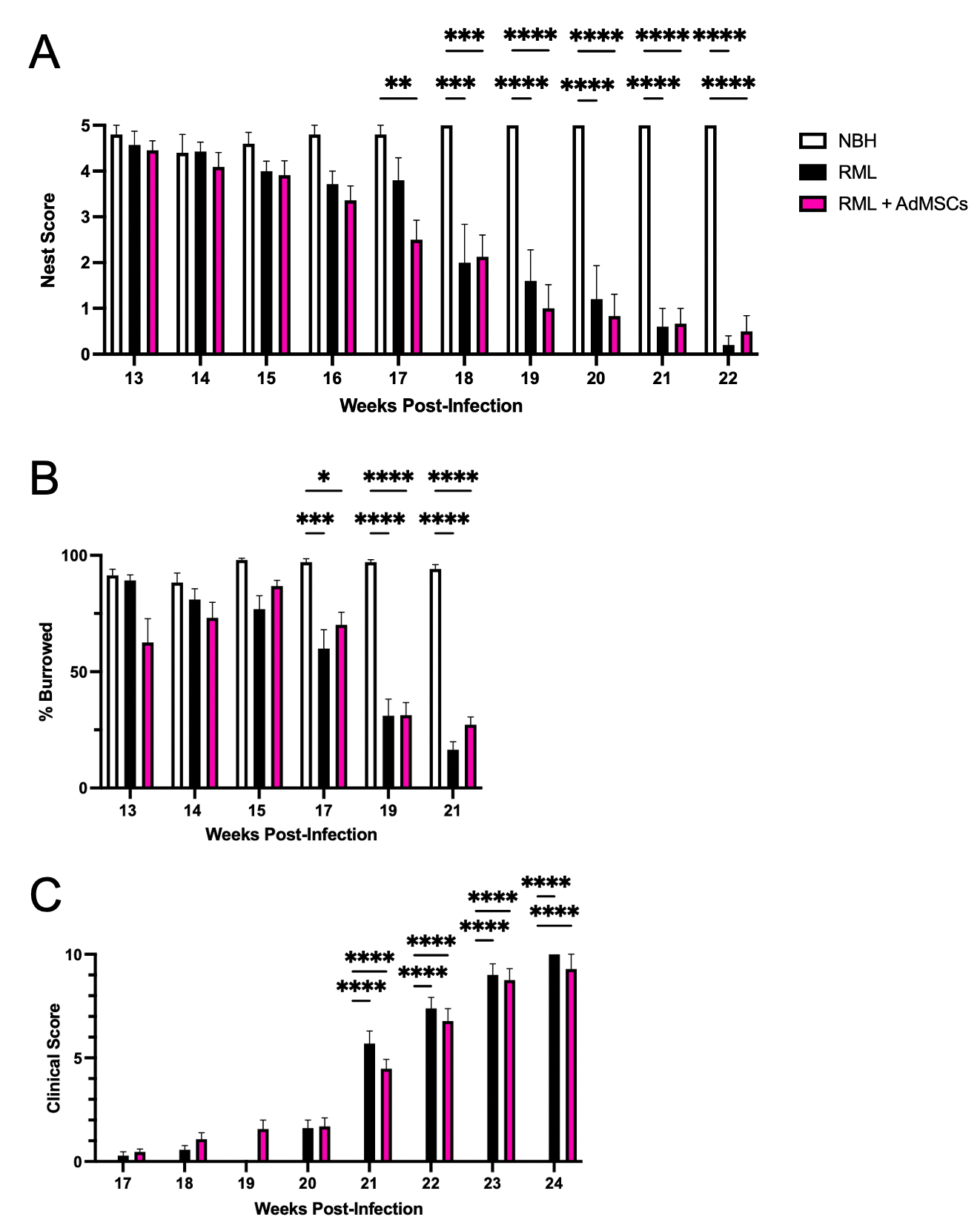


**
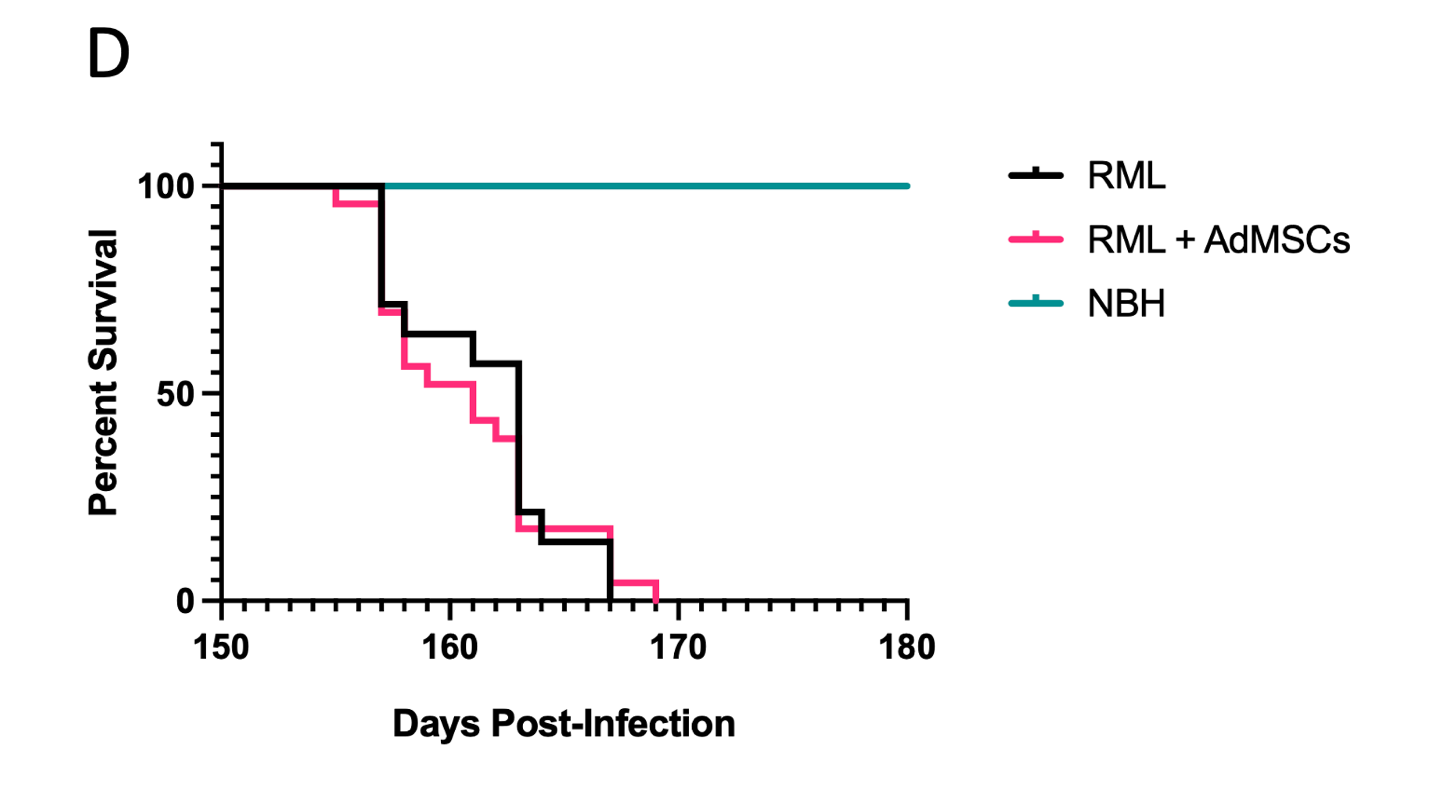
**

## Supplemental Figure 2. Intranasal delivery of AdMSCs did not affect behavioral, clinical signs or survival of prion-infected mice. (A) Mice were analyzed weekly for their ability to build nests and (B) bi-weekly for their ability to burrow beginning at 13 wpi. (C) Clinical scoring was performed weekly beginning at 17wpi. (D) Mice were euthanized after scoring a 10 or higher. Median survival was 163 days for both vehicle and AdMSC-treated animals. Survival curves were compared using a log-rank test. Behavioral and clinical signs were compared between groups using a Two-way ANOVA with post-hoc Tukey’s test, *p<0.05, **p<0.01, ***p<0.001, ****p<0.0001, error bars = SEM.

**
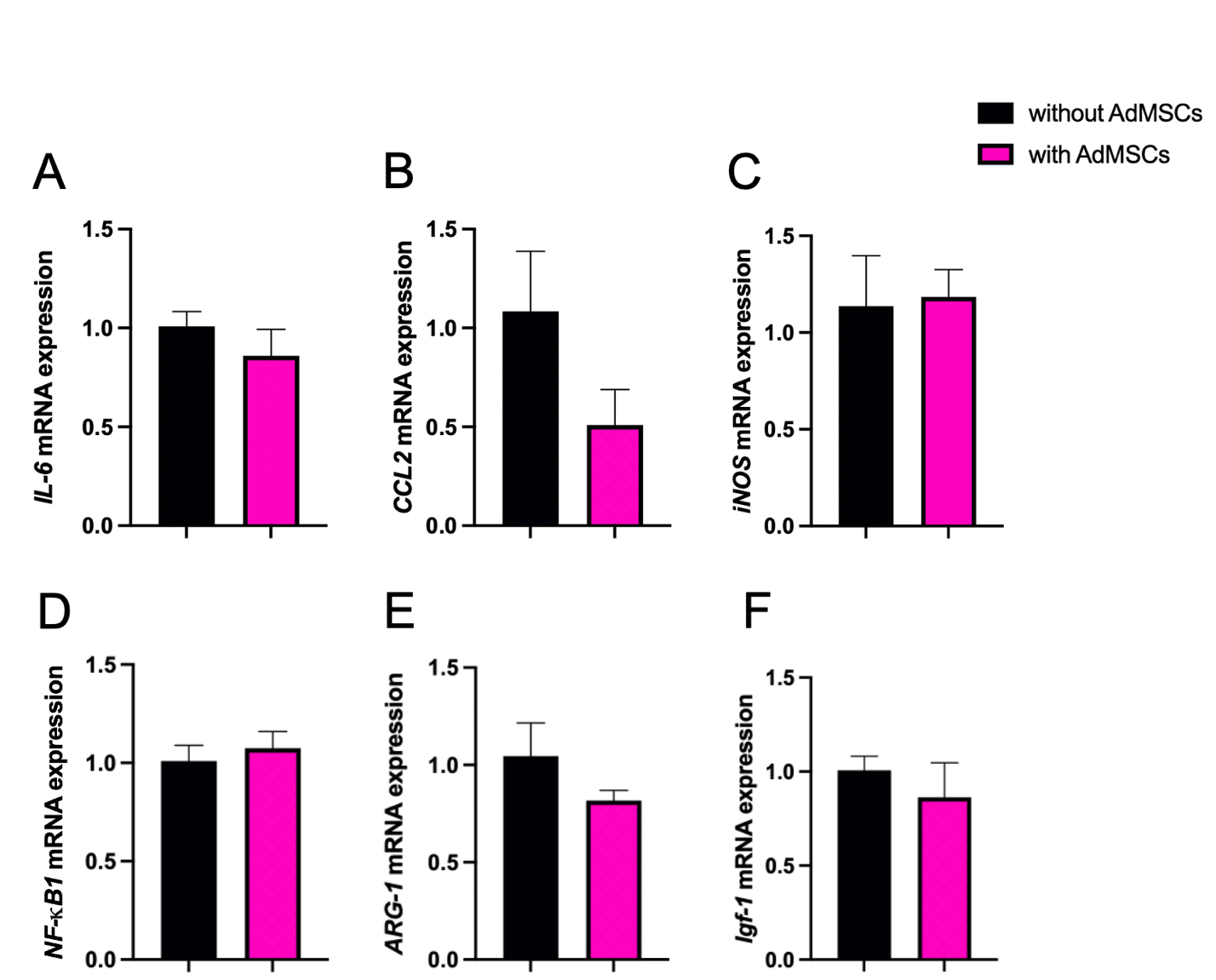
**

## Supplemental Figure 3. AdMSC treatment does not induce changes in some inflammatory cytokine transcripts in the hippocampus at 16 weeks post infection (wpi). No changes were seen in hippocampal mRNA expression for (A) *IL6*, (B) *CCL2*, (C) *iNos*, (D) *NF-κB1*, (E) *Arg-1*, or (F) *Igf-1* with AdMSC treatment. Hippocampi were analyzed from 10 animals, 6 AdMSC-treated and 4 PBS-treated controls. T-test with Welch’s corrections, *p<0.05, **p<0.01, error bars = SEM.

**
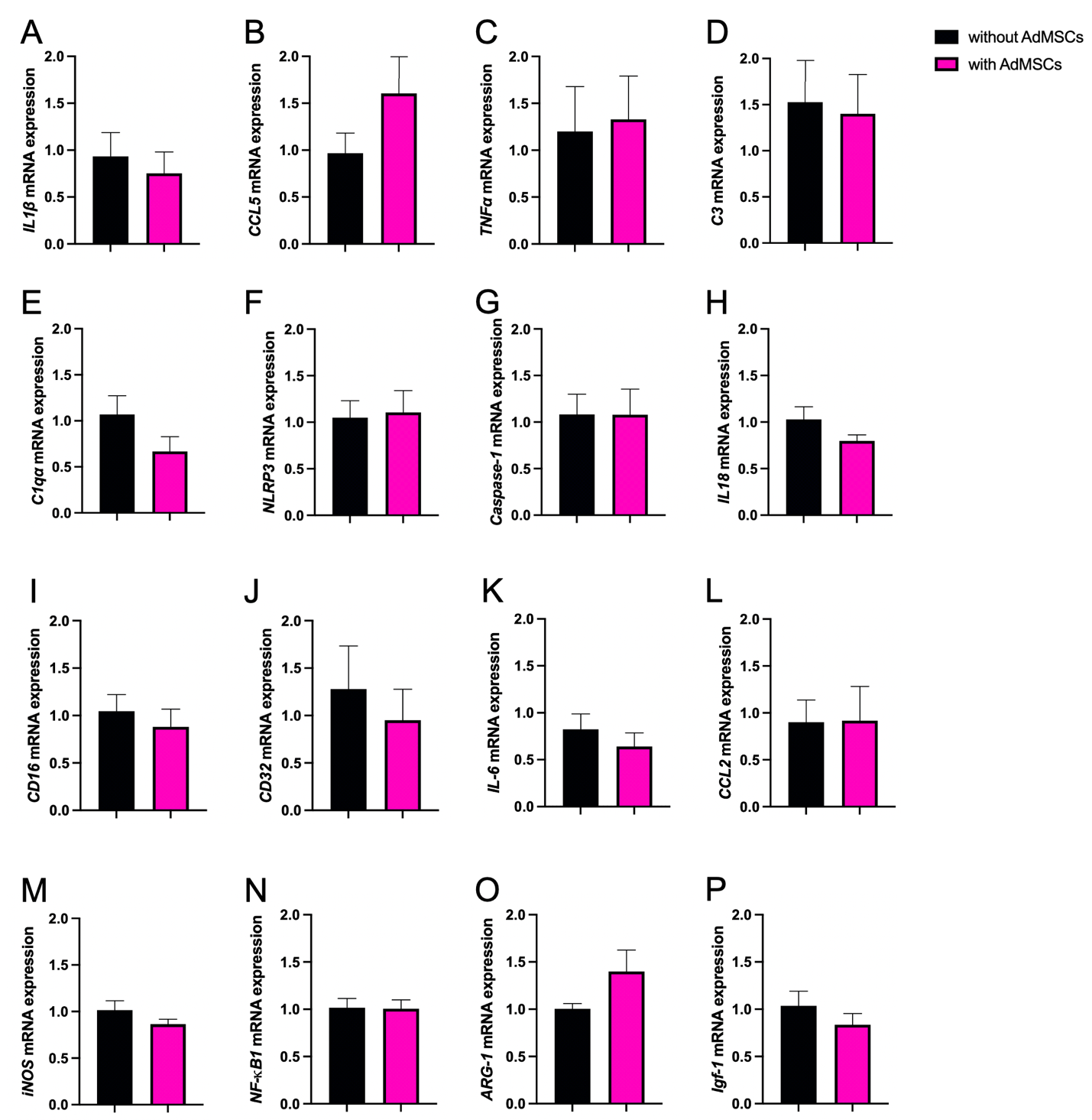
**

## Supplemental Figure 4. AdMSC treatment has no effect on mRNA for inflammatory cytokine transcripts in the hippocampus at 18weeks post infection (wpi). Hippocampal mRNA expression did not change in animals treated with AdMSCs compared to controls for (A) *IL1β*, (B) *CCL5*, (C) *TNFα*, (D) *C3*, (E) *C1qa*, (F) *NLRP3*, (G) *Caspase-1*, (H) *IL18*, (I) *CD16*, (J) *CD32*, (K) *IL6*, (L) *CCL2*, (M) *iNos*, (N) *NF-κB1*, (O) *Arg-1*, or (P) *Igf-1.* Hippocampi were analyzed from 10 animals, 6 AdMSC-treated and 4 PBS-treated controls. T-test with Welch’s corrections, p<0.05, error bars = SEM.

**
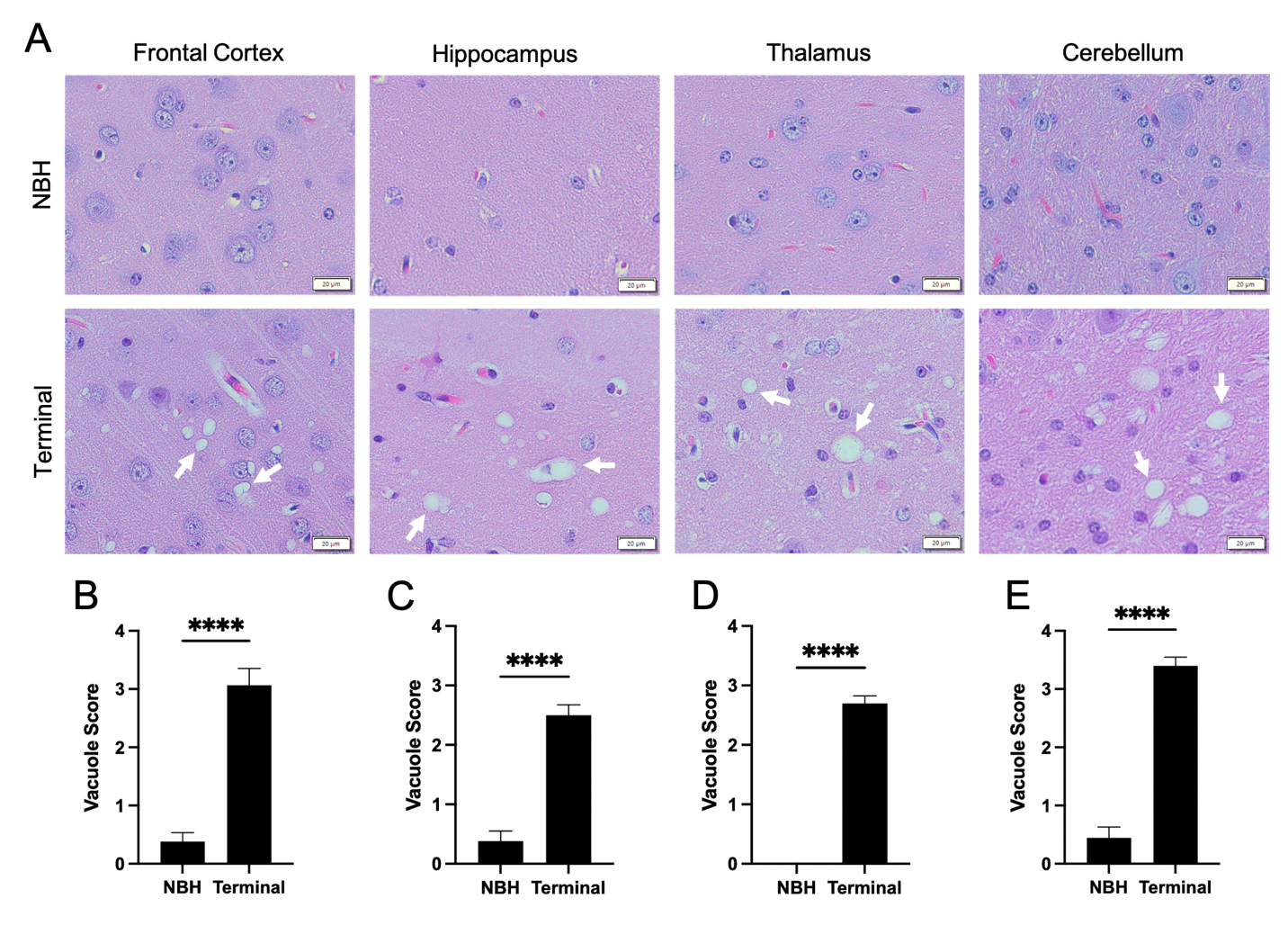
**

**Supplemental Figure 5. Prion-infected mice show significant spongiosis throughout the brain at terminal stages.** (A) H&E stained brains from NBH and terminal mice. Vacuoles in the (B) frontal cortex, (C) hippocampus, (D) thalamus and (E) cerebellum were scored on a scale of 0-5 based on size and number. An average of three scores for each brain region was calculated for each animal. Terminal mice had significantly more vacuoles in all brain regions compared to age-matched controls. H&E stained brains were analyzed from 7 NBH animals and 10 terminal animals. T-test with Welch’s corrections, ****p<0.0001, error bars = SEM. 40x representative images, scale bar = 20 μm.

**
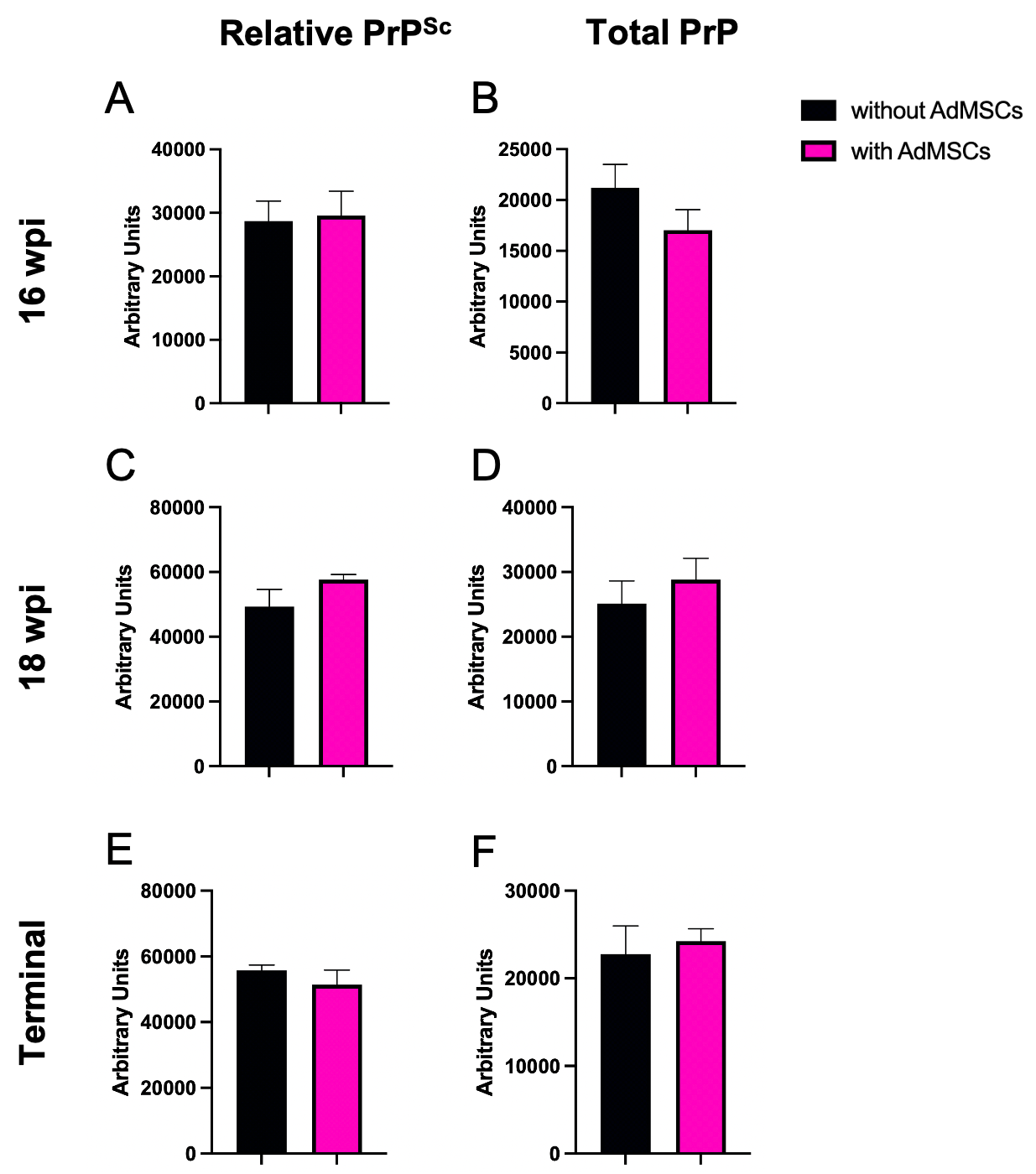
**

## Supplemental Figure 6. AdMSC treatment does not induce detectable changes in PrP^Sc^. Western blots were used to compare both PrP^Sc^ (PK-resistant PrP) and total PrP (not PK-treated) between vehicle and AdMSC-treated mice. At 16 wpi, no difference was detected between (A) PrP^Sc^ or (B) total PrP between treatment groups. At 18 wpi, no difference was detected between (C) PrP^Sc^ or (D) total PrP, and at terminal stages of disease, no difference was detected between (E) PrP^Sc^ or (F) total PrP between treatment groups. See Figure 5 for images of western blots. Brain homogenates were analyzed from 10 animals per timepoint, 6 AdMSC-treated and 4 PBS-treated controls (for terminal mice, 7 AdMSC-treated and 3 PBS-treated controls). T-test with Welch’s corrections, p<0.05, error bars = SEM.

**
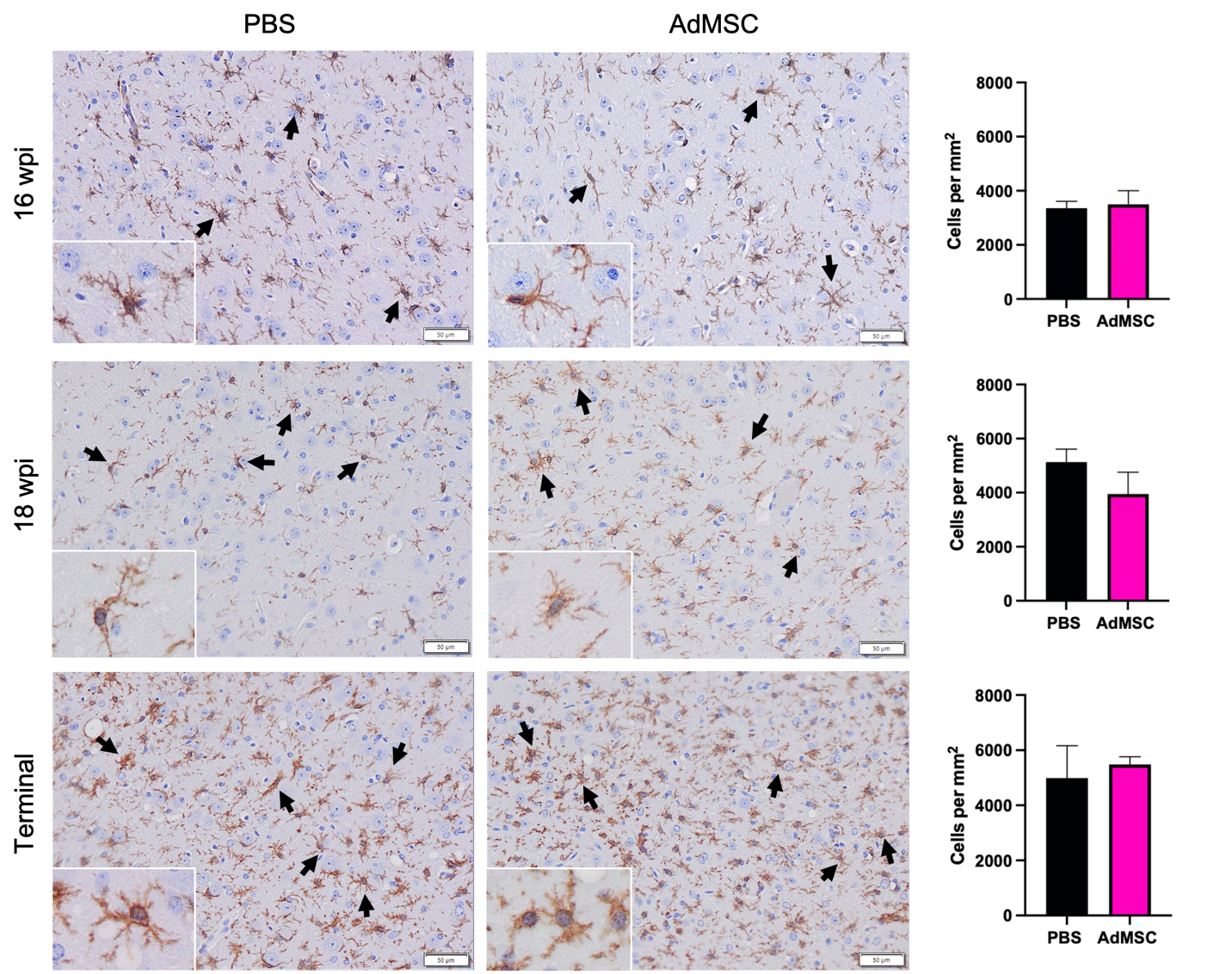
**

**Supplemental Figure 7. AdMSC treatment does not change number of microglia in the thalamus.** Counts of Iba1+ cells were performed in the thalamus. Thalamic microglia were analyzed from 10 animals per timepoint, 6 AdMSC-treated and 4 PBS-treated controls. T-test with Welch’s corrections, error bars = SEM. IHC: 20x, scale bar = 50 μm.

**
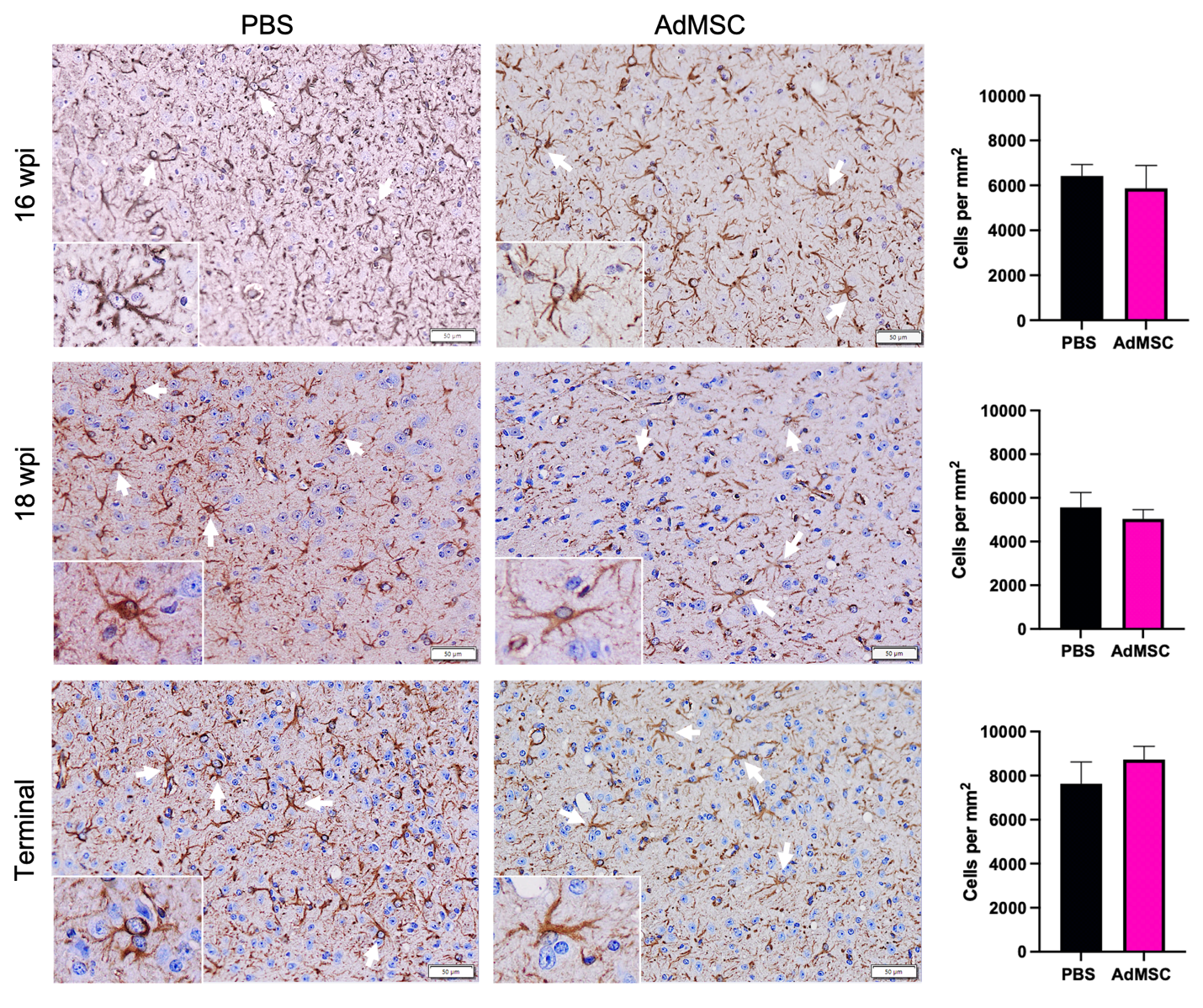
**

**Supplemental Figure 8. AdMSC treatment does not change number of astrocytes in the thalamus.** Counts of GFAP+ cells were performed in the thalamus. Thalamic astrocytes were analyzed from 10 animals per timepoint, 6 AdMSC-treated and 4 PBS-treated controls. T-test with Welch’s corrections, error bars = SEM. IHC: 20x, scale bar = 50 μm.
